# Comparative analysis of the complete genome of KPC-2-producing *Klebsiella pneumoniae* Kp13 reveals remarkable genome plasticity and a wide repertoire of virulence and resistance mechanisms

**DOI:** 10.1186/1471-2164-15-54

**Published:** 2014-01-22

**Authors:** Pablo Ivan Pereira Ramos, Renata Christina Picão, Luiz Gonzaga Paula de Almeida, Nicholas Costa B Lima, Raquel Girardello, Ana Carolina P Vivan, Danilo E Xavier, Fernando G Barcellos, Marsileni Pelisson, Eliana C Vespero, Claudine Médigue, Ana Tereza Ribeiro de Vasconcelos, Ana Cristina Gales, Marisa Fabiana Nicolás

**Affiliations:** 1 Laboratório Nacional de Computação Científica, Petrópolis, Rio de Janeiro, Brazil; 2 Instituto de Microbiologia Paulo de Góes, Universidade Federal do Rio de Janeiro, Rio de Janeiro, Brazil; 3 Departamento de Biologia Geral, Universidade Estadual de Londrina, Paraná, Brazil; 4 Disciplina de Infectologia, Universidade Federal de São Paulo, São Paulo, Brazil; 5 Departamento de Patologia Clínica, Análises Clínicas e Toxicologia, Universidade Estadual de Londrina, Paraná, Brazil; 6 Laboratoire d’Analyse Bio-informatique en Génomique et Métabolisme CNRS-UMR 8030, Commissariat à l’Energie Atomique (CEA), Institut de Génomique, Genoscope, Evry, France

**Keywords:** Carbapenemase, Comparative genomics, *Enterobacteriaceae*, Gram-negative, Nosocomial pathogens, Pathogenic bacteria, SNPs

## Abstract

**Background:**

*Klebsiella pneumoniae* is an important opportunistic pathogen associated with nosocomial and community-acquired infections. A wide repertoire of virulence and antimicrobial resistance genes is present in *K. pneumoniae* genomes, which can constitute extra challenges in the treatment of infections caused by some strains. *K. pneumoniae* Kp13 is a multidrug-resistant strain responsible for causing a large nosocomial outbreak in a teaching hospital located in Southern Brazil. Kp13 produces *K. pneumoniae* carbapenemase (KPC-2) but is unrelated to isolates belonging to ST 258 and ST 11, the main clusters associated with the worldwide dissemination of KPC-producing *K. pneumoniae*. In this report, we perform a genomic comparison between Kp13 and each of the following three *K. pneumoniae* genomes: MGH 78578, NTUH-K2044 and 342.

**Results:**

We have completely determined the genome of *K. pneumoniae* Kp13, which comprises one chromosome (5.3 Mbp) and six plasmids (0.43 Mbp). Several virulence and resistance determinants were identified in strain Kp13. Specifically, we detected genes coding for six beta-lactamases (SHV-12, OXA-9, TEM-1, CTX-M-2, SHV-110 and KPC-2), eight adhesin-related gene clusters, including regions coding for types 1 (*fim*) and 3 (*mrk*) fimbrial adhesins. The *rmtG* plasmidial 16S rRNA methyltransferase gene was also detected, as well as efflux pumps belonging to five different families. Mutations upstream the OmpK35 porin-encoding gene were evidenced, possibly affecting its expression. SNPs analysis relative to the compared strains revealed 141 mutations falling within CDSs related to drug resistance which could also influence the Kp13 lifestyle. Finally, the genetic apparatus for synthesis of the yersiniabactin siderophore was identified within a plasticity region. Chromosomal architectural analysis allowed for the detection of 13 regions of difference in Kp13 relative to the compared strains.

**Conclusions:**

Our results indicate that the plasticity occurring at many hierarchical levels (from whole genomic segments to individual nucleotide bases) may play a role on the lifestyle of *K. pneumoniae* Kp13 and underlie the importance of whole-genome sequencing to study bacterial pathogens. The general chromosomal structure was somewhat conserved among the compared bacteria, and recombination events with consequent gain/loss of genomic segments appears to be driving the evolution of these strains.

## Background

*Klebsiella pneumoniae* is a non-motile, rod-shaped, Gram-negative bacterium belonging to the *Enterobacteriaceae* family. It occupies diverse ecological niches ranging from soil to water, but from an anthropocentric perspective it represents one of the most important human pathogens [1,2]. *K. pneumoniae* are commonly reported as etiologic agents of either community-acquired urinary tract infections or bacterial pneumonia. However, it can cause any type of infection in hospital settings, including outbreaks in newborns and adults under intensive care, which is likely associated to its ability to spread rapidly in the hospital environment [1].

A wide repertoire of virulence and resistance factors is present in *K. pneumoniae* genomes, allowing for the expression of capsule, siderophores, adhesins and antimicrobial resistance determinants [3,4].

At present, six complete *K. pneumoniae* subsp. *pneumoniae* genomes are available on the public databases, namely *K. pneumoniae* strains MGH 78578 (multidrug-resistant [MDR] bacterium isolated from a patient with pneumonia [5]), NTUH-K2044 (a hypermucoviscosity (HV)-positive isolate obtained from a patient with liver abscess and meningitis [3]), 342 (N_2_-fixer plant endophyte still bearing pathogenic potential against mice [4]), HS11286 (MDR clinical isolate [6]), KCTC 2242 (a 2,3-butanediol producer [7]) and 1084 (an HV-negative clinical isolate causing liver abscess [8]). Other 115 on-going *K. pneumoniae* sequencing projects are available as scaffolds/contigs at the NCBI Genome database (http://www.ncbi.nlm.nih.gov/genome/genomes/815). The availability of closed genomes is important towards understanding the *K. pneumoniae* genome plasticity and the evolutionary forces that drive their genetic differentiation and occupation of distinct ecological niches. For instance, Wu et al. [3] showed that the methionine salvage pathway, which might have a role in pathogenesis via oxidative stress, is present in all *Klebsiella* genomes but not in *Escherichia*, *Salmonella* or *Shigella*, thus providing diversity in virulence mechanisms in pathogenic enterobacteria. Moreover, from the comparative genomic analyses between strains 342 and MGH 78578, Fouts et al. [4] identified variations in the distribution of genes related to surface attachment, regulation and signaling, secretion and transport, all of which may have important implications concerning their preferred lifestyle and host ranges (endophytic plant associations for 342 and human pathogen for MGH 78578).

Among the antimicrobial resistance repertoire of *K. pneumoniae*, the production of carbapenemase is particularly worrisome since it confers resistance to all beta-lactams. KPC (*Klebsiella pneumoniae* carbapenemase) is the main Ambler’s class A carbapenemase found in this species, representing an emerging public health issue in many countries like USA, Greece, Israel, and Brazil [9]. Interestingly, it has been shown that the worldwide dissemination of KPC-producing *K.pneumoniae* occurred due to the expansion of ancestrally-related isolates comprising the Clonal Complex 11 (CC11). Even though representatives of KPC producing CC11 have also successfully disseminated in Brazil, many KPC-2 producers reported in that country are not related to this epidemic lineage [10,11].

In May 2009, *K. pneumoniae* isolate Kp13 (hereafter referred as Kp13) was obtained from the blood culture of a patient admitted to the intensive care unit (ICU) of a teaching hospital located in the city of Londrina, Southern Brazil. Kp13 was one of the representative strains of a clone that caused a nosocomial outbreak in that hospital that involved 18 ICU patients. It was the first time KPC producers were identified in that healthcare institution. Of notice, nearly 60% of cases died in average within 18 days after KPC-producing *K. pneumoniae* isolate was recovered. Although this outbreak was due to the clonal dissemination of KPC-producing isolates, Kp13 showed a distinguished antimicrobial susceptibility profile, expressively more resistant than the other isolates (Eliana C. Vespero, personal communication).

In addition, it was previously reported that Kp13 possesses a unique capsular gene cluster, *cps*_Kp13_, among the approximately 80 capsular serotypes described to date [12]. The genome of Kp13 was fully sequenced and showed to be constituted by one chromosome and six plasmids. To our best knowledge, it represents the first available complete and closed genome of a *K. pneumoniae* isolated from Latin America, as well as the first one of a *K. pneumoniae* responsible for causing a large nosocomial outbreak.

In this report, we perform a genomic comparison between Kp13 and three *K. pneumoniae* genomes (MGH 78578, NTUH-K2044 and 342), to gain insights into the resistance and virulence repertoire of this strain, its unique/shared gene content relative to the compared strains and plasticity regions that may be circulating in other clinically relevant bacteria.

## Results and discussion

### General genomic features of *K. pneumoniae* isolate Kp13

MLST analysis classified Kp13 under the ST 442, sharing a single alelle with both ST 258 and ST 11, the main clusters associated with the worldwide dissemination of KPC-producing *K. pneumoniae*. Of notice, clinical isolates belonging to ST 442 have been only identified in Brazil so far [10,11].

**Figure 1:**
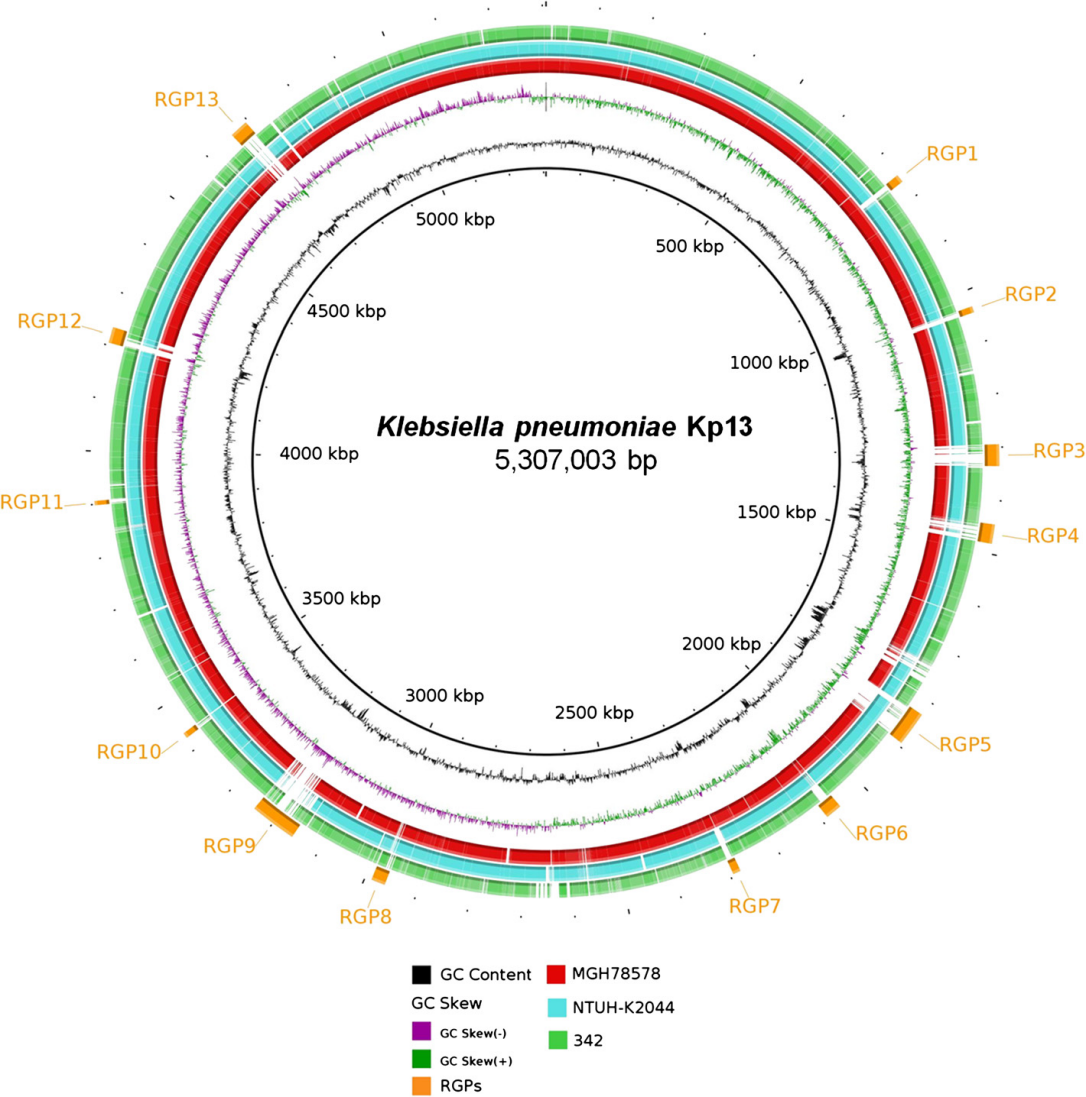
**Circular map of the chromosome of *K. pneumoniae *Kp13 and related bacteria.** The innermost ring represents the *K. pneumoniae* Kp13 chromosome used as reference and its coordinates. The second ring (in black) plots the G + C content of the reference, followed by its G + C skew (in purple/green). Red, cyan and green rings that follow depict BLASTN comparisons between the chromosomes of Kp13 and those of strains MGH 78578, NTUH-K2044 and 342, respectively. The outermost, interspaced rings (in orange) represent the localization of the predicted regions of genomic plasticity in the Kp13 chromosome, and the labels of each region follow the ones in Table 2.

The complete genome of *K. pneumoniae* isolate Kp13 consists of 5,739,888 bp of which 5,307,003 bp form a single, circular chromosome (Figure 1) and 432,885 bp are split into six plasmids which range in size from 2,459 bp (pKP13a) to 294,493 bp (pKP13f) (Table 1). All the replicons were completely closed and the putative coding sequences (CDS) were manually validated. The reported sequence data can be obtained from the NCBI (http://www.ncbi.nlm.nih.gov/bioproject) under project accession ID PRJNA78291.

**Table 1:** **Main features of the *K. pneumoniae *** **Kp13 genome compared to related bacteria**

**Organism**	**Size (bp)**	**G + C content (%)**	**No. of CDSs**^**‡**^	**Mean CDS size (bp)**	**Coding density (%)**	**No. of tRNAs**	**Reference**
*K. pneumoniae* Kp13							
**Chromosome**	5,307,003	57.5	5,288	896	89.3	86	This study
**Plasmids**							
pKP13a	2,459	49.2	3	553	67.5	0	
pKP13b	3,223	56.3	5	505	78.4	0	
pKP13c	5,065	43.8	9	350	62.3	0	
pKP13d	45,574	46.0	63	590	81.6	0	
pKP13e	81,071	51.1	108	636	84.8	0	
pKP13f	295,493	47.9	365	682	84.2	0	
*K. pneumoniae* NTUH-K2044							
**Chromosome**	5,248,520	57.7	5,130	939	89.4	86	[3]
**Plasmids**							
pK2044	224,152	50.2	270	696	83.4	0	
*K. pneumoniae* MGH 78578							
**Chromosome**	5,315,120	57.5	4,776	958	86.1	86	Washington University
**Plasmids**							
pKPN3	175,879	51.7	178	756	76.5	0	
pKPN4	107,576	53.4	123	726	83.1	0	
pKPN5	88,582	53.8	98	694	76.8	0	
pKPN6	4,259	41.4	5	551	64.7	0	
pKPN7	3,478	45.7	5	343	49.3	0	
*K. pneumoniae* 342							
**Chromosome**	5,641,239	57.3	5,425	915	88.0	88	[4]
**Plasmids**							
pKP187	187,922	47.2	230	608	74.5	0	
pKP91	91,096	51.1	113	635	78.8	0	

^‡^Number of CDSs according to the submitted GenBank flatfile downloaded from NCBI for each studied genome.

The G + C content of the Kp13 chromosome is 57.5%, which is in agreement with values previously reported for NTUH-K2044 (57.7%), MGH 78578 (57.5%) and 342 (57.3%) (Table 1). However, the plasmidial sequences present a much lower G + C content of 49.1 ± 4.4% (mean and standard deviation for the six replicons) suggesting the existence of DNA acquired during events of horizontal gene transfer (HGT), an observation that is corroborated by the number of mobile genetic elements (MGEs) such as transposons and phage sequences found in these plasmids. The number of transfer RNAs (tRNAs) identified in Kp13 was 86, which is identical to those of MGH 78578 and NTUH-K2044, but different than that reported in the strain 342 that carries 88 tRNAs genes (Table 1). Regarding the chromosome size, Kp13 lies between the human pathogens NTUH-K2044 and MGH 78578 [3], while *K. pneumoniae* 342 presents the largest chromosome size (Table 1). This is probably due to the accumulation of genes related to its adaptation to a different ecological niche since this strain is a N_2_-fixing bacterium [4]. However, when considering only plasmidial sequences, the DNA content of Kp13 (432,885 bp, six plasmids) is markedly higher than other compared strains being followed by MGH 78578 (379,774 bp distributed among its five plasmids, Table 1). In fact, as it will be further discussed in this paper, a number of Kp13’s virulence- and resistance-related genes are plasmid-mediated and their respective proteins may contribute for evasion of host defenses and survival under antimicrobial selective pressure.

### Comparison of the chromosomal architecture between *K. pneumoniae*

The overall architecture of the chromosomes of *K. pneumoniae* Kp13, NTUH-K2044, MGH 78578 and 342 were studied using the Mauve tool [13], and these multiple alignments are shown in Additional file 1. This analysis evidenced an overall structural conservation and colinearity among the chromosomes of the different *K. pneumoniae* strains compared. The multiple alignments showed the existence of nine large locally colinear blocks (LCBs); however, it was observed that strain-specific regions were also present within LCBs (white segments in Additional file 1), which may harbor specific adaptations of each bacterium, as it will be further discussed in the plasticity segment section. These regions most likely represent DNA acquired during events of HGT and may provide a greater metabolic versatility to Kp13 strain [14]. For instance, the chromosome of N_2_-fixing strain 342 was the one that most accumulated differences relative to other human pathogens (Additional file 1), such as the *nif* cluster located in a plasticity region unique to that strain (KPK_1696-KPK_1715) [4].

Chromosomal inversions are thought to be one of the main events of genomic rearrangements in bacteria [15]. Of notice, no recombination events leading to inversions of DNA segments occurred within the larger LCBs (Additional file 1). Only LCB2 and LCB4 (both smaller than 10 kbp) were found inverted relative to the Kp13 chromosome (Additional file 2). This observation reinforces the conserved chromosomal architecture among the compared *K. pneumoniae* strains since these strains are not epidemiologycally related and, in the case of 342, can be considered ecologically distinct from one another.

### Regions of genomic plasticity between Kp13 and other *K. pneumoniae*

A total of 13 RGPs were identified in Kp13’s chromosome when compared to the chromosomes of 342, MGH 78578 and NTUH-K2044 (Figure 1, Table 2). Most of these regions contain sequences related to transposable elements, and were flanked by genes coding for tRNAs or phage-structures (Table 2), which can be regarded as evidences for their horizontal acquisition [16]. The amount of DNA located in these regions accounts for at least 400.1 kbp or 7.5% of the Kp13 chromosome. The main characteristics of these RGPs are next discussed, but additional information is provided as supporting information (see Additional file 3).

**Table 2:** **Regions of Genomic Plasticity (RGPs) identified in the *K. pneumoniae *** **Kp13 chromosome**

**Region**	**Coordinates**	**Length (kbp)**	**No. of CDSs**	**G + C%**	**Features**	Best hit (% coverage, e-value)*
RGP1	748,751 – 764,155	15.4	17	59%	s.b.; dGC%; transposases	*K. pneumoniae* MGH 78578 (100%, 0.0)
RGP2	1,031,543 – 1,043,427	11.9	9	37%	s.b.; dGC%; phage; transposases	*Enterobacter cloacae* ATCC 13047 (10%, 0.0)
RGP3	1,294,343 – 1,335,472	41.1	55	52%	s.b.; dGC%; phage	*Escherichia coli* IHE3034 (23%, 0.0)
RGP4	1,444,954 – 1,481,531	36.6	36	50%	s.b.; dGC%; T6SS; tRNA; transposases	*Salmonella enterica* (55 %, 0.0)
RGP5	1,827,686 – 1,893,227	65.5	59	53%	s.b.; tRNA; dGC%; T4SS	*Enterobacter hormaechei* (93%, 0.0)
RGP6	2,056,825 – 2,088,335	31.5	35	52%	s.b.; T6SS; dGC%; transposases	*K. pneumoniae* NTUH-K2044 (85%, 0.0)
RGP7	2,278,838 – 2,293,758	14.9	17	46%	s.b.; dGC%; transposases	*K. pneumoniae* HS11286 (91%, 0.0)
RGP8	2,961,023 – 2,987,614	26.6	20	60%	s.b.; T6SS; dGC%	*K. pneumoniae* KCTC 2242 (98%, 0.0)
RGP9	3,155,750 – 3,244,119	88.4	111	52%	s.b.; dGC%; phage	*K. pneumoniae* MGH 78578 (29%, 0.0)
RGP10	3,425,935 – 3,436,504	10.6	9	48%	s.b.; dGC%; transposases	*K. pneumoniae* MGH 78578 (75%, 0.0)
RGP11	3,898,258 – 3,906,459	8.2	9	54%	s.b.; transposases	*K. pneumoniae* MGH 78578 (100%, 0.0)
RGP12	4,204,889 – 4,234,405	29.5	33	48%	s.b.; dGC%; tRNA; transposases	*K. pneumoniae* KCTC 2242 (43%, 0.0)
RGP13	4,660,071 – 4,695,371	35.3	32	50%	s.b.; dGC%; tRNA; transposases	*K. pneumoniae* KCTC 2242 (60%, 0.0)

*BLASTN performed against the ‘nr’ database at the NCBI website; dGC%, deviation from mean chromosomal GC content; s.b., synteny break from at least one other compared *K. pneumoniae*; phage, probable bacteriophage region; tRNA, presence of flanking transfer RNAs; transposases, presence of transposase-related sequences; T4SS/T6SS, presence of, respectively, types IV/VI secretion systems.

**Figure 2:**
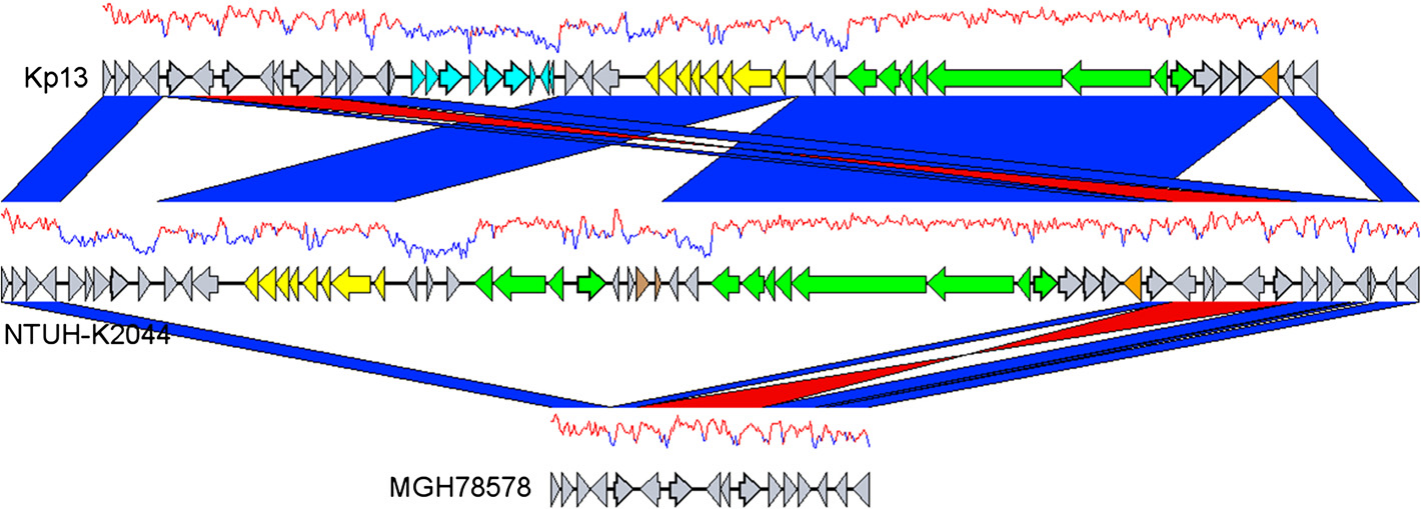
**Genomic context of RGP5 (*Yersinia *high-pathogenicity island) detected in Kp13.** Arrows represent predicted genes and their respective transcription strands (forward/reverse). In yellow, those that code for T4SS proteins; in blue, genes unique to Kp13; in green, genes related to siderophore production and export. The central segment unique to NTUH-K2044 contains the *iro* cluster related to iron capture. In orange and brown are shown, respectively, integrases and transposases. Plots above each region indicates the corresponding G + C content (%). The vertical blue and red segments connecting regions represent, respectively, direct or inverted conserved segments (BLASTN ≥ 70%).

RGP1 carries an insertion sequence at its 5′-end (IS*1* family, KP05176) and harbors CDSs with predicted catalytic domains at the protein level including two possible dioxygenases (KP02716, KP02718) and a dehydrogenase (KP02715), which may be involved in the degradation of aromatic compounds [17], although further studies will be necessary to confirm this role. RGP2 contains genes for transposases and a possible phage integrase (KP16268) as well as hypothetical genes which do not allow for any inference on a possible metabolic role. RGP3 is 41 kbp in length and represents a phage insertion in the Kp13 chromosome, with the majority of its CDSs sharing greater identity to those of *Salmonella* phage SPN1S [GenBank:NC_016761]. A second, large bacteriophage-related RGP was detected in the Kp13 chromosome (RGP9, Table 2) and part of this region exhibits remarkable sequence similarity to that of *K. oxytoca ϕKO2* prophage [18]. Apart from phage sequences, a possible antibiotic resistance cluster that contains genes coding for transcriptional regulators (KP04065, KP04061), an antibiotic biosynthesis monooxygenase (KP04063) and a glyoxalase/bleomicin dioxygenase (KP04064) were found, but further experimental studies are warranted to establish the relevance of this region to the MDR phenotype displayed by Kp13. RGP4 is one of three plasticity segments containing genes responsible for type VI secretion system (T6SS) formation. T6SS proteins are part of a recently described apparatus that secretes toxins using a needle-like mechanism [19]. They have been previously implicated in bacterial pathogenesis such as the HSI-I locus of *Pseudomonas aeruginosa* associated with persistent infections by this bacteria [20]. A CDS coding for the VgrG protein was identified in RGP4 (KP01061). This protein may play both the role of an effector (influencing the target cell physiology) as well as of structural component of the T6SS where it has been reported to form part of the puncturing needle [21]. Two other T6SS-related RGPs were detected in the Kp13 chromosome inside of the RGP6 and RGP8. RGP6 contains CDSs coding for bacteriocins, proteins that target other bacteria and may provide competitive advantage to Kp13. In contrast, the third T6SS locus found in RGP8 harbors the *hcp* gene (KP04341) whose product, Hcp, is also part of the needle structure along with VgrG [21]. It has been shown that Hcp inhibited phagocytosis by macrophages in *Aeromonas hydrophila*, thus acting as an effector in modulating the host’s innate immune response [22,23]. A noteworthy plasticity region detected in the Kp13 chromosome was located in RGP5 (Figure 2), which has been previously established in other bacteria as being an ICE (Integrative and Conjugative Element) [24]. The integration of this region into the Kp13 chromosome could have been facilitated by tRNAs (for asparagine) located at its flanking termini as has been shown for other *K. pneumoniae*[24]. Genes usually involved in plasmidial mobilization (*mobC*, KP04798 and *mobB*, KP04799) as well as in T4SS (KP04803-KP04813) are also present and could have aided in the mobilization of this region (Figure 2). A great part of the gene composition of RGP5 is related to that of *Yersinia pestis* high-pathogenicity island (HPI) which is present in many *Enterobacteriaceae* genera [24]. The presence of genes coding for the yersiniabactin siderophores (*irp1,* KP04825 and *irp2,* KP04826) are characteristic of this HPI, and their occurrence in more virulent *K. pneumoniae* strains has been previously reported [24]. The presence of this region is indicative that Kp13 is well adapted as a pathogen, since iron scavenging is important during infection, as this nutrient is poorly available in the host in its free form. Alternative siderophore systems were also found in the Kp13 genome and will be further detailed. The ICE region is absent in the clinical strain MGH 78578 as well as in the N_2_-fixing strain 342 that harbors in this region a different 115 kbp insertion containing genes related to its symbiotic lifestyle. Compared to NTUH-K2044 each strain carries unique segments within this RGP: in Kp13, this unique region is 12 kbp-long and contains several uncharacterized genes (KP04788-KP04791) (Figure 2). Since this ICE is usually found in more virulent strains, the search for this region in other *Enterobacteriaceae* strains isolated in Brazil may help to estimate its frequency and importance in clinically relevant bacteria. RGP7 represents an insertion found only in the chromosomes of Kp13 and MGH 78578 and contains CDSs with conserved domains at their predicted protein sequence such as glycosyltransferase (KP05060), acyltransferase (KP05301), two-component histidine kinase sensor (KP05061, KP05062) and peptidase (KP05059). The presence of *kexD* (KP05076) in this RGP called for special attention, since this gene codes for the recently characterized multidrug efflux pump KexD, an inner membrane protein belonging to the RND family [25]. It was found to be overexpressed in a number of multidrug-resistant *K. pneumoniae* isolates, and confered resistance to erythromycin, tetracycline, ethidium bromide and other drugs [25]. RGP12 contains Kp13-specific fimbriae and adhesins genes (KP02150-KP02154) and this insertion occurs after a tRNA gene for threonine. The *aco* operon (*acoKABCD*, KP32150; KP02898-KP02901) involved in acetoin catabolism [26] was also found in this region. It was detected in all compared strains, except for MGH 78578.

The large number of RGPs detected in Kp13 relative to the strains MGH 78578, NTUH-K2044 and 342 indicate a complex pattern of gain and loss of genetic material segments that occurred during the shaping of each of the compared chromosomes throughout their evolutionary history. It is thought that such events occur more frequently in pathogenic than in non-pathogenic bacteria, the genomes of the first group being regarded as more unstable and rearrangement-prone [27].

### Unique and shared genes between the compared *K. pneumoniae*

We have analyzed the group of shared and unique genes between Kp13, MGH 78578, NTUH-K2044 and 342 to obtain insights that might explain their distinct virulence and pathogenic features.

**Figure 3:**
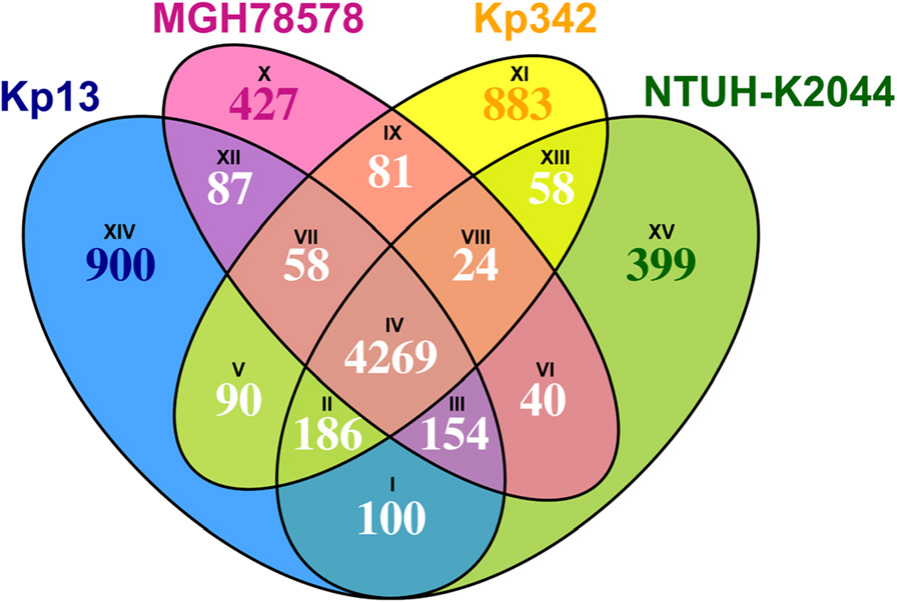
**Unique and shared gene content among the genomes of *K. pneumoniae *Kp13, NTUH-K2044, MGH 78578 and 342.** The roman numbers at each partial intersection correspond to the CDSs in Additional file 4.

The results from this analysis are shown in Figure 3 and in Additional file 4. As expected, the majority of the coding sequences from the compared organisms are part of a conserved genomic ‘core’ comprising 4,269 CDSs (Figure 3). Of these, 98.8% are chromosomally located in Kp13. As the chromosomes are generally more stable replicons, it is not surprising that most of the conserved genes were detected in this genetic replicon. Most of the so-called ‘accessory’ genes, which are present or not in bacterial strains, were found in plasmids of the compared *K. pneumoniae*. In Kp13, 40% of its unique genes (region XIV in Figure 3) were encoded in plasmids. The largest part of these unique genes (693 CDSs) could not have any functional clues attributed and were annotated as ‘hypothetical’ (Additional file 4), highlighting the need for follow-up studies focusing on whether these genes are in fact expressed and their possible roles.

Among the shared genes found only in clinical isolates (region III in Figure 3) we detected CDSs whose products are related to phosphonate utilization (*phn* cluster, KP00091 and adjacent genes in Kp13). These were previously studied in *Klebsiella* and may provide means to use alternative phosphorus sources such as aminoethylphosphonate, ethylphosphonate and methylphosphonate [28]. A CDS coding for D-aminoacylase (*dan*, KP32221) was also detected, and shared 49% identity with the recently characterized protein from *Achromobacter xylosoxidans* subsp. *denitrificans* (BLASTP against [Swiss-Prot:P72349]). This enzyme is involved in the conversion of *N*-acyl-D-amino acids to D-amino acids and fatty acids [29]. CDSs annotated as sulfatases were also found only in the clinical *K. pneumoniae* studied (Kp13 loci KP03498, KP03499), and their products may play a role in sulphate utilization.

Most of the unique genes found in Kp13 (region XIV in Figure 3) are related to MGEs such as transposases, insertion sequences and phage genes (Additional file 4). There are also CDSs whose products are involved in capsule formation (*cps* cluster) such as glycosyltransferases (KP03791, KP03802 and KP03803), which are unique to Kp13. The structure of this cluster was previously studied and belonged to a hitherto unreported capsular serotype [12]. CDSs whose predicted proteins have similarity to fructosamine deglycase (KP01294, 40% BLASTP identity to *Bacillus subtilis* [Swiss-Prot:O32157]) and fructosamine kinase (KP01295, 43% identity to [Swiss-Prot:O32153]) were also unique to Kp13. These enzymes are usually involved in the utilization of Amadori products, and fructosamine itself is produced via the condensation of glucose with amine compounds [30]. We formulated the hypothesis that, if indeed the products of these CDSs play a role in fructosamine metabolism as energy source, they could provide the Kp13 isolate with an adaptive advantage in the nosocomial setting, since Amadori products are intimately related to advanced glycation end-products (AGEs). In turn, AGEs are produced as result of poorly controlled chronic diseases such as diabetes [31], and from this perspective these compounds could enter the bacterial cell through an adjacent transporter coded in CDS KP01297 and be converted into glucose by the aforecited enzymes, providing Kp13 with an alternative energy source within the compromised host. In fact, Kp13 was recovered from a blood culture of a diabetic patient. However, additional studies, using animal models, are required to establish the occurrence of increased frequency and/or exacerbation of Kp13-infection in hyperglycemia. Another set of unique genes found in Kp13 included the cassettes *sat* (KP32246, located in plasmid pKP13d), coding for streptothricin acetyltransferase involved in the resistance to streptothricin antibiotics in the context of a class 2 integron, and a CDS whose product may be related to dihydrofolate reductase activity (KP31590, located in plasmid pKP13f within a class 1 integron), which may confer resistance to trimethoprim. The latter will be further discussed in the context of *bla*_CTX-M-2_.

### Virulence- and resistance-related ‘weapons’ identified in Kp13

#### *Siderophores*

Iron scavenging is important within the mammalian host environment since its free form is usually found at very low concentrations under physiological conditions [32]. Iron-acquisition systems were previously studied in other strains including NTUH-K2044 and have been associated with increased virulence [33,34]. The list of siderophore systems found in Kp13 is presented in Additional file 5.

We have identified genes coding for proteins responsible for the conversion of chorismic acid into enterobactin (*entABEC* cluster, KP03396 and adjacent CDSs) as well as for the transport of this siderophore, which is ubiquitously found among enterobacteria (*fep* cluster, KP31892 and adjacent CDSs). The FhuABCD system was also found in Kp13 (coded by CDSs KP01825-KP01828) and is involved in the uptake of ferrichrome, a fungal siderophore [35]. We have also found the genetic apparatus needed for yersiniabactin synthesis, which was previously discussed on the context of the plasticity region RGP5. Another siderophore receptor homolog (the product of CDS KP04902) displaying 70% identity to the aerobactin receptor IutA (BLASTP against [SwissProt:P14542]) was found, and could also represent a way for Kp13 to incorporate exogenous chelating compounds since the *iucABCD* cluster was not present in this strain, although it was found in the plasmid of NTUH-K2044 (coded in CDS pK2044_01340 and adjacencies). Other siderophore-independent iron uptake systems in Kp13 include the *hmuRSTUV* cluster (KP02540-KP02544), which probably code for an ABC family heme transporter; the *feo* (ferrous iron transport) cluster, which synthesizes three proteins (KP31748, KP00683, KP00684) involved in Fe^2+^ capture; and the *sitABCD* system, also from the ABC family of proteins involved in the transport of divalent cation such as Mn^2+^ and Fe^2+^; found in CDSs KP02550-KP02553, respectively. The diverse repertoire of iron uptake system detected in Kp13 likely influence its ability to cause infection and survive within the human host.

#### *Adhesins*

The expression of adhesins is important during the colonization stage when diverse mechanical forces such as peristalsis and salivary secretion all act to hamper bacterial invasion within the host [36]. The list of adhesins detected in *K. pneumoniae* Kp13 and related bacteria is found in Additional file 6. Most of the genes coding for these proteins were found in all *K. pneumoniae* strains compared, except for a fimbrial cluster present in a previously discussed plasticity region (RGP12).

Type 1 fimbriae coding genes were detected in Kp13, including the *fim* cluster (KP02223 and adjacent genes). Their products were previously reported as enhancing virulence during urinary tract infection caused by *K. pneumoniae*[37]. The *mrk* cluster composed by five genes (*mrkABCDF,* Additional file 6) that code for type 3 fimbriae is also present in Kp13. In *K. pneumoniae*, type 3 fimbriae have been identified as accessories mediating the formation of biofilms on biotic and abiotic surfaces such as catheters in the hospital environment [38].

Apart from type 1 (*fim*) and type 3 (*mrk*) fimbrial adhesins, we also identified in Kp13 six other clusters related to the expression of these virulence factors which were previously studied in strain NTUH-K2044 [39]: *kpa* (KP00426-KP00430), *kpb* (KP00500-KP00503), *kpd* (KP03577-KP03580), *kpe* (KP05219-KP05222), *kpf* (KP02456-KP02459) and *kpg* (KP02460; KP05178; KP04093). As has been previously observed [39], strain 342 lacks the *kpf* cluster which was found in the clinical isolates and is composed by genes coding for a fimbrial subunit (KP02456), a chaperone (KP02457), an usher protein (KP02458) and an adhesin (KP02459). Since strain 342 occupies a distinct ecological niche, further studies are warranted to determine the implications of *kpf* expression to the pathogenesis of clinical *K. pneumoniae*.

The identification of different adhesin clusters, including one previously unreported, should be important for the control of Kp13 and related pathogenic bacteria, as these proteins have been already proposed as good vaccine candidates [40].

#### Enzymatic inactivation of antimicrobials

The resistance determinant *bla*_SHV_ gene, which codes a narrow-spectrum class A beta-lactamase known to confer the intrinsic resistance to amoxicillin, ampicillin, ticarcillin and carbenicillin, was identified in Kp13 [41]. Indeed, both *K. pneumoniae* MGH 78578 and NTUH-K2044 genomes contain a *bla*_SHV-11_ copy, encoding the narrow-spectrum SHV variant. Kp13 showed two copies of *bla*_SHV_ genes: *bla*_SHV-12_ (found in the plasmid pKP13f) encoding an extended-spectrum beta-lactamase (ESBL); and a chromosomally-encoded *bla*_SHV-110_ gene coding for an enzyme whose spectrum of activity has not been characterized yet (Table 3). The *bla*_SHV-12_ copy was located downstream an IS*26* element (KP05944), while *bla*_SHV-110_ was found downstream the *lac* operon and was followed by an uncharacterized HTH-type transcriptional regulator CDS (KP01127).

**Table 3:** Characteristics of the beta-lactamases identified in the Kp13 genome

**Product**	**Gene**	**CDS**	**Genetic element**	Best hit (% identity*, isolation country)
Beta-lactamase SHV-12	*bla*_SHV12_	KP32248	pKP13f	*K. pneumoniae* MGH 78578 (100%, USA)
Beta-lactamase OXA-9	*bla*_OXA9_	KP01389	pKP13f	*K. pneumoniae* FC1 (100%, Argentine)
Beta-lactamase TEM-1	*bla*_TEM-1_	KP01391	pKP13f	*Neisseria gonorrhoeae* (100%, nd)
Beta-lactamase CTX-M-2	*bla*_CTX-M-2_	KP03128	pKP13f	*Salmonella typhimurium* CAS-5 (100%, Argentine)
Beta-lactamase SHV-110	*bla*_SHV110_	KP31849	Chr	*K. pneumoniae* FSP 237/05 (100%, Brazil)
Beta-lactamase KPC-2	*bla*_KPC-2_	KP06703	pKP13d	*K. pneumoniae* 15 (100%, USA)

*,BLASTP against the NCBI database; Chr, chromosome; nd, not determined; pKP13[d/f], plasmid pKP13d/pKP13f.

**Figure 4:**
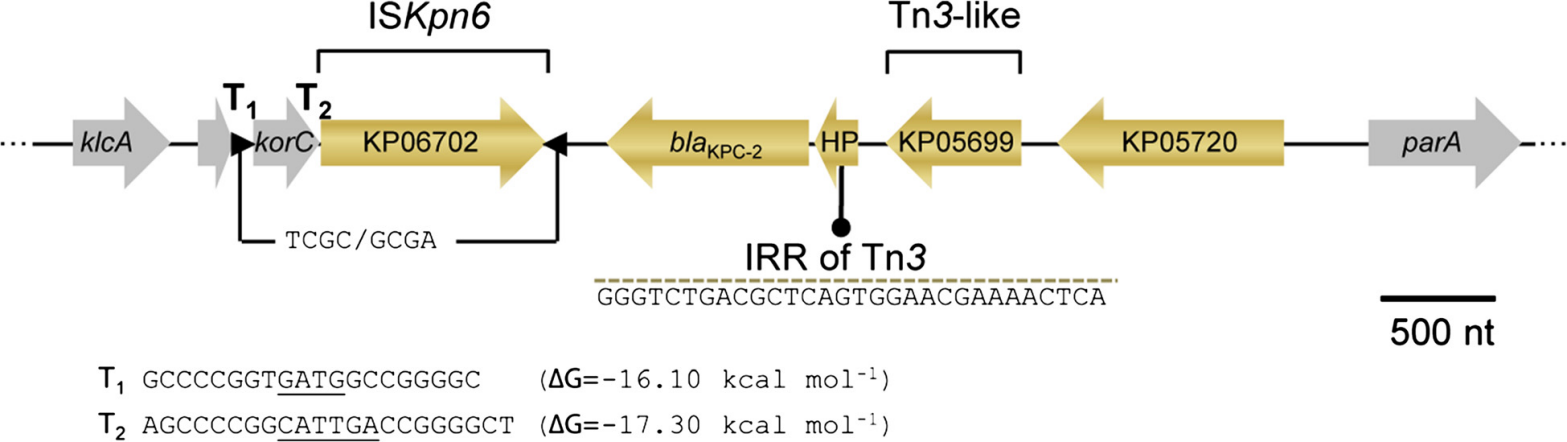
**Genetic context of *bla***_**KPC-2 **_**in *K. pneumoniae *Kp13.** Genes are depicted as horizontal arrows (orientations indicate their transcriptional direction). Some structures of this region are highlighted, such as an inverted repeat right (IRR) of the Tn*3* transposon, as well as inverted repeats flanking IS*Kpn6* (depicted as black triangles). Also shown are the localization of two rho-independent transcriptional terminators (T_1_, T_2_) identified using the ARNold webserver [49] and their corresponding sequences.

Several acquired beta-lactamase-encoding genes were identified in plasmids pKP13f and pKP13d of Kp13, namely *bla*_TEM-1_, *bla*_OXA-9_, *bla*_CTX-M-2_, and *bla*_KPC-2_ (Table 3), genes that if properly expressed are enough to confer resistance to all beta-lactam agents. *bla*_TEM-1_ encodes a class-A beta-lactamase with limited spectrum of activity, that confers resistance against penicillins and first-generation cephalosporins and whose activity is inhibited by clavulanic acid [42]. The *bla*_OXA-9_ gene probably encodes a class-D beta-lactamase with narrow spectrum of activity, although its complete biochemical characterization remains to be determined. OXA-9 is inhibited by clavulanic acid and cloxacillin, but differently from most OXA enzymes is not inhibited by NaCl, conferring a phenotype typical of class A enzymes production [43]. The *bla*_CTX-M-2_ gene encodes an ESBL commonly observed in *Enterobacteriaceae* isolates from humans, swine and poultry manure, although this gene has also been identified in *P. aeruginosa*, *Vibrio cholerae* and *Acinetobacter baumannii* clinical isolates [44]. In Kp13, *bla*_CTX-M-2_ is found downstream a class 1 integrase CDS (KP03119) and adjacent to the Orf513-coding gene (KP03126), also named ISCR1, which possibly mediates the dissemination of this gene [45]. As previously described [45], this integron contains a duplication of its 3′ segment where truncated *qacE* (between CDSs KP18328 and KP03130) and *sul1* (KP03130 and KP03134) were found. The overall architecture of the *bla*_CTX-M-2_ region in Kp13 is closer to that described for the class 1 integron harboring *bla*_CTX-M-59_ (termed In*0506*) recovered from patients in São Paulo, Brazil [46], with which the Kp13 sequence shares 99% identity (BLASTN against GenBank accession no. EU622856), and the difference relies on the non-synonymous change in *bla*_CTX-M-2_. This region also carries resistance determinants to other antimicrobial classes, including the genes *dfrXVb* (KP31590), encoding an alternative dihydrofolate reductase that is associated with resistance to trimethoprim and a *cmlA-*like (KP18323), which codes for an efflux pump related to chloramphenicol resistance [47]. Of special interest in Kp13 was the detection of *bla*_KPC-2_ (KP06703, found in pKP13d) gene coding for the *K. pneumoniae* class A carbapenemase (KPC-2). The expression of KPC-2, especially when associated with porin alterations, can give rise to resistance against all beta-lactams. The genetic context of *bla*_KPC-2_ includes an upstream insertion sequence (IS*Kpn6*, KP06702), as well as a resolvase CDS (Figure 4)*.* It is of notice that no inverted repeats related to Tn*4401* were detected, since this transposon is frequently associated with the mobilization of the carbapenemase gene [48]. The absence of such signals can be regarded as evidence that the spread of *bla*_KPC-2_ also occurs solely by recombination events involving IS*Kpn6* and the flanking Tn*3*-family sequences.

#### Resistance by target alteration

Mutations in specific sites in genes coding for proteins used as targets for antimicrobials, such as GyrA and ParC, can result in decreased drug sensitivity. Kp13 showed the double substitutions Ser83 → Phe and Asp87 → Asn in GyrA (KP00955) which are known to confer ciprofloxacin resistance [50]. Also, a single amino acid change, Ser80 → Ile, was detected in ParC (KP02784), and the combined effect of these mutations could have contributed to the high-level resistance to quinolones displayed by Kp13 as has been shown for other pathogenic bacteria [51]. MGH 78578 carries the Ser83 → Tyr mutation in GyrA, while in NTUH-K2044 no mutations in this gene were detected. No changes in the ParC quinolone resistance-determining region were detected in these strains.

Another well recognized resistance mechanism by target alteration is the expression of 16S rRNA methyltransferases. These enzymes transfer methyl radicals to certain nitrogenous bases located at the A site of 16S rRNA in the 30S portion of the ribosome and confers resistance to most clinically-relevant aminoglycosides [52]. Kp13 harbored the *rmtG* gene (KP01427) in plasmid pKP13f, which encodes a 16S rRNA methyltransferase recently described from a *K. pneumoniae* clinical isolate from Brazil [53]. Immediately upstream this gene a putative tRNA ribosyltransferase CDS (KP01428) was found. A CDS annotated as a transposase (KP01425) is located close to the 3′-end of *rmtG,* and search against the ISFinder database classified this sequence in the IS*91* family, although it is unclear whether this transposon is involved in the mobilization of *rmtG*.

#### Efflux pumps, porin alterations and polymyxin resistance

We searched the genome of Kp13 and related *K. pneumoniae* for known MDR efflux pumps. Table 4 summarizes these results, which include pumps belonging to the five main families of efflux proteins. The *tolC* gene coding for accessory protein TolC used by many systems is found in CDS KP02796 in Kp13.

**Table 4:** **Drug-related efflux pumps identified in compared *K. pneumoniae***

**System**	**CDS**^**∆**^	**Resistance profile**^**Ω**^	**Presence in**			
			**Kp13**	**NTUH-K2044**	**MGH 78578**	**342**
*RND family*						
AcrAB	KP03628	AC, BL, BS, CM, CV, EB, FA, FQ, ML, NO, OS, RF, SDS, TX	+	+	+	+
AcrD	KP03833	AG, DC, FU, NO	+	+	+	+
KexD	KP05076	EM, EB, NO, RD, TPP	+	−	+	−
MdtABC	KP03148	BS, DC, NO	+	+	+	+
OqxAB	KP02440	CM, FQ, NA, SDS	+	+	+	+
*MF family*						
Bcr	KP04395	BI, STZ	+	+	+	+
EmrAB	KP31576	CCC, NA, TCS, TLM	+	+	+	+
Fsr	KP03612	FM	+	+	+	+
MdfA (KdeA)	KP04269	AG, BENZ, CM, EB, FQ, RF, TC	+	+	+	+
MdtG	KP04943	DC, FOM	+	+	+	+
MdtH	KP04933	ENX (FQ), NFX	+	+	+	+
MdtL	KP32205	CM	+	+	+	+
SmvA	KP04515	CV, EB	+	+	+	+
*SMR family*						
EmrE	KP32244	AC, CV, EB	+	−	−	−
SugE	KP00509	BENZ, EB	+	+	+	+
*MATE family*						
MdtK	KP05135	AC, NFX	+	+	+	+
*ABC family*						
MacAB	KP04225	ML	+	+	+	+
MdlAB	KP03663	?	+	+	+	+

∆, in the case where the pump is encoded by multiple CDSs, the ID corresponds to one of the adjacent CDSs. Ω, based in the literature found for each system. Substrate nomenclature adapted from [54]: AC, acriflavine; AG, aminoglycosides; BI, bicyclomycin; BL, beta-lactams; BS, bile salts; BENZ, benzalkonium chloride; CCC, carbonyl cyanide chlorophenylhydrazone; CM, chloramphenicol; CV, crystal violet; DC, deoxycholate; EB, ethidium bromide; EM, erythromycin; ENX, enoxacin; FA, fatty acids; FQ, fluoroquinolones; FOM, fosfomycin; FM, fosmidomycin; FU, fusidic acid; ML, macrolides; NA, nalidixic acid; NFX, norfloxacin; NO, novobiocin; OS, organic solvents; RD, rhodamine; RF, rifampicin; SDS, sodium dodecyl sulfate; STZ, sulfathiazole; TC, tetracycline; TCS, tetrachlorosalicylanilide; TLM, thiolactomycin; TPP, tetraphenylphosphonium chloride; TX, Triton X-100; ?, regarded as related to multidrug-resistance, but with an unknown resistance spectrum. *, evaluated by BLASTP against the target genome.

One of the main efflux-related resistance mechanisms involves the expression of the *acrAB* genes, coding for a periplasmic protein (AcrA, KP03629) and a transporter protein (AcrB, KP03630) which use the aforecited TolC channel. Expression of these genes is regulated by the adjacent *acrR* gene (KP03628), as well as by *marR, ramR* and *soxR* genes; mutations in these genes may result in *acrAB* overexpression, thus contributing to the MDR phenotype [55]. However, we did not detect alterations in these genes, suggesting that other efflux systems in Kp13 might be involved in the extrusion of antimicrobials (Table 4). Another well-studied efflux pump identified in Kp13 and other compared *K. pneumoniae* strains is OqxAB (coded in CDSs KP02440/KP02441), which was first identified in *E. coli* plasmids. It has been widely reported in *K. pneumoniae* clinical isolates, where it is usually chromosomally located [56]. The expression of *oqxAB* has been associated with resistance to quinolones [57], and it remains to be investigated whether this system contributes to the high-level fluoroquinolones resistance displayed by Kp13. Other efflux systems found in the compared *K. pneumoniae* include homologs of AcrD (RND family), MdfA and MdtH (both from the MF superfamily) (Table 4), involved in the efflux of aminoglycosides and fluoroquinolones [58], compounds to which Kp13 displays resistance. The majority of the efflux pumps are conserved among the different strains and most of them can recognise more than one substrate (Table 4). Despite this qualitative conservation, we have identified several non-synonymous mutations in various genes coding efflux pumps in the Kp13 genome when compared with NTUH-K2044 and MGH 78578 (see SNPs analysis section and the table in Additional file 7), although whether these alterations change the specificity profile of these systems remains an open question.

We studied the common porins present in *K. pneumoniae* bacteria, namely OmpK35 (homolog to OmpF in *E. coli*) and OmpK36 (the OmpC homolog). In strain Kp13, the genes coding for these proteins are found in CDSs KP04180 and KP00950, respectively. While we observed an overall conservation at the gene sequence level between Kp13 and the compared strains for both *ompK35/ompK36*, a peculiarity in the chromosome of Kp13 in the region upstream *ompK35* was detected with the insertion of an IS*1380* family transposase (KP04179), which does not occur in the compared *K. pneumoniae* strains. Multiple alignment of this region and *in silico* promoter search suggested that this recombination event disrupted the probable promoters needed for *ompK35* expression (see Additional file 8 [A]). Experimental evidence corroborating this hypothesis was obtained by the extraction and analysis of outer membrane (OM) proteins using SDS-PAGE [59], where OmpK35 was not visualized (Additional file 8 [B]). Porin loss was previously studied in *K. pneumoniae* and the non-expression of *ompK35* was associated with increased resistance to antibiotics such as cephalosporins and cephamycins [60]. Thus, loss of OmpK35 appears to be another mechanism employed by strain Kp13 that contributed to its MDR profile.

Polymyxin B is a rapid-acting bactericidal agent that interacts with the OM lipopolysaccharide (LPS) in Gram-negative bacteria. It was recently shown that the two-component systems PhoPQ and PmrAB together with the Rcs system govern polymyxin-induced transcriptional changes, and that there is a cross talk between PhoPQ and the Rcs system [61]. In the genome of Kp13, we identified all these regulatory systems: PhoQP (in CDSs KP04870, KP04869), PmrAB (in CDSs KP03034, KP3033) and RcsBCD (in CDSs KP00953, KP00954, KP00952). Transcriptional studies are ongoing to determine the regulons networks that govern polymyxin B-induced changes in Kp13.

### Interstrain variation at the nucleotide level may relate to lifestyle-specific adaptations

**Figure 5:**
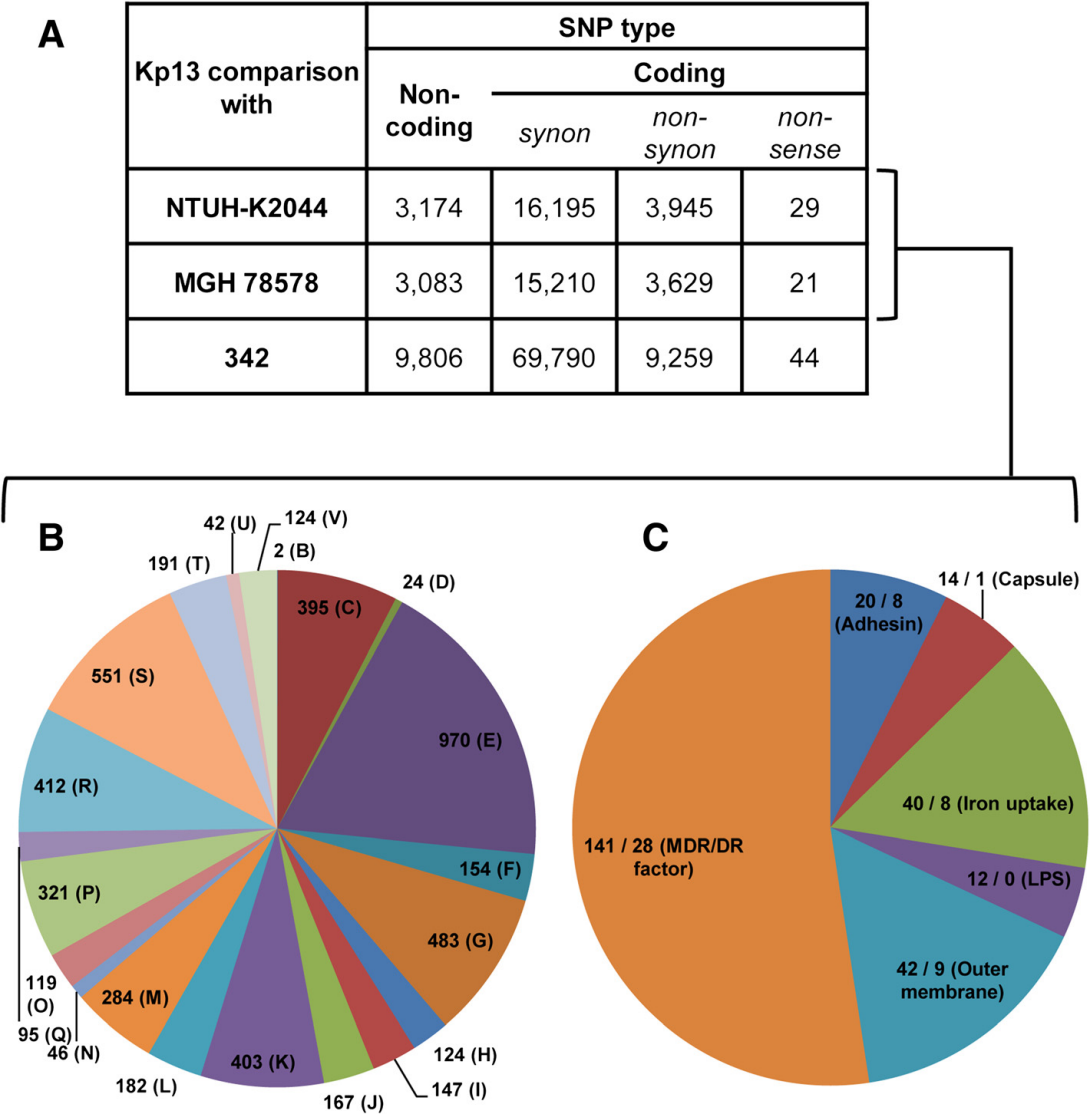
**Single-nucleotide polymorphisms (SNPs) identified in *K. pneumoniae *Kp13 compared to MGH 78578, NTUH-K2044 and 342 strains. (A)** Total number of SNPs falling within non-coding and coding regions (CDSs) when compared with the three other strains. *synon,* synonymous. **(B)** Common SNPs falling within CDSs grouped by COG classification identified when compared with the strains NTUH-K2044 and MGH 78578. **(C)** Selected common SNPs falling within virulence- and resistance-related CDSs identified when compared with the NTUH-K2044 and MGH 78578 strains. *MDR/DR,* multidrug resistance/drug resistance; *LPS,* lipopolysaccharide. Numbers after the slash indicates the number of non-synonymous SNPs. COG classes single-letter abbreviations in panel B are: B (chromatin structure), C (energy production/conversion), D (cell division/chromosome partitioning), E (amino acid transport/metabolism), F (nucleotide transport/metabolism), G (carbohydrate transport/metabolism), H (coenzyme transport/metabolism), I (lipid transport/metabolism), J (translation/ribosomal structure), K (transcription), L (replication/recombination/repair), M (cell wall/membrane/envelope biogenesis), N (cell motility), O (posttranslational modification/protein turnover/chaperones), P (inorganic ion transport/metabolism), Q (secondary metabolite biosynthesis/transport/catabolism), R (general function prediction), S (function unknown), T (signal transduction), U (intracellular trafficking/secretion), V (defense mechanisms).

SNPs were determined for three comparisons using the complete chromosome sequence of Kp13 as reference aligned against: i) the complete chromosome sequence of strain MGH 78578, ii) complete chromosome sequence of strain NTUH-K2044 and iii) complete chromosome sequence of strain 342. The number of SNPs falling within non-coding and coding regions (non-synonymous, synonymous and non-sense) between each comparison are listed in Figure 5A. It can be seen that strain 342 accumulated a total number of mutations much larger than the other two strains in comparison to Kp13 (data not shown). This result is in accordance with the proposed hypothesis that this strain should be reclassified as *K. variicola*[62]. Then, we investigated the common SNPs identified in all CDSs of Kp13 (grouped according to their COG functional category) against both pathogenic strains, MGH 78578 and NTUH-K2044 (Figure 5B and Additional file 9). These SNPs represented unique Kp13 alleles relative to the latter two human pathogenic strains. Disregarding uninformative categories such as general function prediction and unknown function, the ones which accumulated more SNPs were E (amino acid transport/metabolism) and G (carbohydrate transport/metabolism). Also, 124 SNPs were identified within CDSs related to category V (defense mechanisms). Subsequently, in order to understand the significance of SNPs in relation to the Kp13 lifestyle, we investigated all SNPs falling within CDSs related to virulence and resistance. Notably, most SNPs (141 in total) were identified within CDSs involved in drug resistance (MDR/DR) (Figure 5C, Additional file 7). They are followed by CDSs whose products function as OM proteins, accounting for 42 mutations, such as the CDS for an OmpA domain-containing protein, which carries four non-synonymous mutations (out of a total of 9) (Additional file 7). However, the role of non-synonymous mutations found among CDSs involved in drug resistance in *K. pneumoniae* remains unknown.

The SNPs analysis performed in Kp13 relative to the two other human pathogenic strains has shown that there are Kp13-specific alleles occurring in many functional COGs, suggestive that these SNPs could result in a variety of diverse phenotypic effects. Moreover, the amount of SNPs found in CDSs associated with drug resistance may give additional clues on the distinctness of Kp13 compared to MGH 78578 and NTUH-K2044. An interesting follow-up for this study would be to analyze the relationship between the amino acid sequence substitutions in these proteins and their corresponding structural alterations, which could relate to the resistance phenotype of Kp13. As an example, non-synonymous mutations in possible MDR-related genes were detected, such as in *mdtB* (respective changes at the protein level Ile93 → Met, Ala352 → Thr, Arg526 → His, considering the Kp13 CDS KP04638 as reference) and an *acrB* homolog (Thr511 → Ala, Val542 → Ala) (Additional file 7).

Finally, the results from the SNPs analysis shed light on the plasticity of the Kp13 genome at the nucleotide level, which complement the observed plasticity at the broader level of genomic segments, discussed within the context of RGPs.

## Conclusions

We have thoroughly mined the genome of *K. pneumoniae* strain Kp13, which to our best knowledge is the first completely determined and closed genome of a *K. pneumoniae* representative involved in a large hospital outbreak. The Kp13 genome is composed by one chromosome and six plasmids of variable sizes. They harbor multiple resistance and virulence loci, coding a diverse range of siderophores, efflux pumps, beta-lactamases and adhesins that might contribute to the succesfull adaptation of this strain to the hospital environment. We also identified alterations in the porin profile of Kp13, which were confirmed experimentally. Multiple regions of genomic plasticity were detected in this strain including the yersiniabactin high pathogenicity island and T6SS-related loci, which could be propagating through HGT to related bacteria. Although the compared genomes present an overall conserved architecture, we have demonstrated the singular characteristics of Kp13 that differentiates this strain from previously studied *K. pneumoniae* and may have contributed to its fitness and succesfull spread on the hospital environment. Within the context of chromossomal architecture and RGPs analyses, we might also assume that the major force that models the genomes of the compared *K. pneumoniae* strains is HGT instead of genomic rearrangements. Moreover, the genome plasticity of isolate Kp13 was corroborated at multiple levels of analysis, from the broader, whole genome structure context, to the finer level of single-nucleotide polymorphisms.

## Methods

### Ethics statement

The Ethics Committee of the Universidade Estadual de Londrina (UEL) approved the present study under reference number CAAE:3356.0.000.268-09. Clinical evaluation and blood sampling were performed after diagnostic routine procedures in the intensive care unit of the Hospital Universitário (UEL) with written informed consent of the patient.

### Bacterial strain

*Klebsiella pneumoniae* isolate Kp13 was obtained from the blood culture of a patient admitted to the intensive care unit with diabetes mellitus and cranial encephalic trauma [12]. This bacterium was isolated during an event of nosocomial outbreak due to KPC-2-producing *K. pneumoniae* that occurred at the Hospital Universitário (UEL) between February and May 2009. The antimicrobial susceptibility profile of Kp13 was confirmed by broth microdilution. Antimicrobials tested included ampicillin, amoxicillin/clavulanic acid, piperacillin/tazobactam, cefoxitin, ceftriaxone, ceftazidime, cefepime, imipenem, meropenem, ertapenem, gentamicin, amikacin, levofloxacin, polymyxin B and minocycline, and tigecycline. MICs were determined and interpreted following the CLSI guidelines for *Enterobacteriaceae*[63,64] except for tigecycline and polymyxin B where FDA [65] and EUCAST [66] breakpoints were applied, respectively. Kp13 was susceptible to tigecycline (MIC, 0.25 mg/L) and minocycline (MIC, 2 mg/L), and displayed resistance to ampicillin (MIC, >32 mg/L); amoxicillin/clavulanic acid (MIC, >32/16 mg/L), piperacillin/tazobactam (MIC, >128/4 mg/L), cefoxitin (MIC, >32 mg/L), ceftriaxone (MIC, >64 mg/L), ceftazidime (MIC, >32 mg/L), cefepime (MIC, >32 mg/L), ertapenem (MIC, ≥1024 mg/L), imipenem (MIC, 64 mg/L); meropenem (MIC, 64 mg/L), amicacin (MIC, >64 mg/L), gentamicin (MIC, 256 mg/L) and polymyxin B (32 mg/L). In addition, this isolate showed the carbapenemase producer phenotype and was confirmed to carry the gene encoding KPC-2 [12].

### DNA sequencing, genome assembly and annotation

Both shotgun and 3 kb paired-end library were constructed and the genome sequencing of *K. pneumoniae* isolate Kp13 was carried out using the Genome FLX sequencer (454 Life Sciences/Roche), as previously described [12]. Genome assembly was performed using Newbler v 2.6 (Roche) and Celera genome assembly v 6.1 (JCV Institute). Gaps within scaffolds resulting from repetitive sequences were resolved by *in silico* gap filling. We achieved mean sequence coverage of 111× for this genome. The SABIA pipeline [67] was used for gene prediction and automatic annotation followed by manual validation of each predicted CDS.

### MLST analysis

Multilocus sequence typing was performed as previously described [68]. Briefly, the trimmed nucleotide sequences of seven housekeeping genes namely *rpoB*, *gapA*, *mdh*, *pgi, phoE*, *infB* and *tonB* were analyzed using the *Klebsiella pneumoniae* MLST Database, available at http://www.pasteur.fr/recherche/genopole/PF8/mlst/Kpneumoniae.html. For each allele was given a number that combined yielded the sequence type (ST). The goeBURST algorithm was employed to generate a minimum spanning tree using the information of isolates available and the PHYLOViZ tool [69]. Clonal complexes included STs sharing at least 5 identical alleles between their representatives.

### Comparative genomic analyses

The MicroScope platform [70,71] provided some of the tools used for the comparative genomic analyses, such as the *RGPFinder* and *Phyloprofile* modules for determination of regions of genomic plasticity (RGPs) and unique/shared gene content identification, respectively. RGPs are defined as DNA segments over 5 kbp possibly related to events of horizontal exchanges, and they are identified using a series of constraints such as G + C% deviation, compositional biases as measured by the tools Alien_Hunter [72] and SIGI-HMM [73], synteny breaks and proximity to tRNAs. In these analyses, the Kp13 chromosome was set as the reference and RGPs were searched relative to those of strains 342, MGH 78578 and NTUH-K2044. Each identified RGP was manually inspected based on its genomic context and conserved genes located at the flanks of the region. Elements characteristic of horizontal transference events such as tRNAs, transposases and prophage sequences were also searched for within each region. EasyFig [74] was used to generate comparative figures of specific regions, while the BLAST Ring Image Generator 0.95 [75] was used to plot the chromosome map as well as arrange and visualize the RGPs within the overall Kp13 chromosome context.

In order to identify unique and shared gene content, the PhyloProfile Exploration tool within MaGe was used, and the following criteria from Jenssen et al. [76] were adopted to define orthology between two translated open reading frames: (i) the smaller protein covering at least 80% the length of the larger (minLrap ≥ 0.8); (ii) protein identity ≥ 35%. A Venn diagram of the unique/shared gene content was generated with a custom R script using the package VennDiagram [77].

Chromosomal architecture comparison between *K. pneumoniae* strains were carried out using the *progressiveMauve* algorithm implemented in Mauve 2.3.1 [78] at its default parameters. For this analysis, sequences from the chromosome of strains 342 [GenBank:CP000964], MGH 78578 [GenBank:CP000647] and NTUH-K2044 [GenBank:AP006725] were downloaded from the NCBI website and used as input to Mauve.

Phage-related sequences were searched using PHAST [79], while T4SS-related sequences were scanned for using the AtlasT4SS database [80].

The investigation of the resistance and virulence repertoire was facilitated by BLASTX sequence comparison to the Antibiotic Resistance Genes Database [81], as well as throughout the manual annotation of the Kp13 genome, when CDSs related to these functions were flagged. Insertion sequences were characterized using the ISFinder database (http://www-is.biotoul.fr/) [82].

SNP analysis was carried out as previously described [83]. Firstly, three primary SNP-call sets were generated from comparisons between Kp13 and the strains MGH 78578, NTUH-K2044 and 342. Then, scripts in PERL were written to function as quality filters to acquire the most reliable SNP-call sets for the comparisons. Alignments were made using the NUCmer algorithm from the MUMmer v.3.0 package [84]. SNPs were also classified regarding their location on intergenic region or CDS (with its corresponding annotated function and related COG category) [83].

### Nucleotide sequence accession numbers

The NCBI BioProject accession of *K. pneumoniae* Kp13 is PRJNA78291. The sequences reported here have been deposited in the GenBank database (http://www.ncbi.nlm.nih.gov/Genbank) under accession number [GenBank: CP003999] for the chromosome. The six plasmids are available under accession numbers [GenBank:CP003996] (pKP13a), [GenBank: CP003994] (pKP13b), [GenBank: CP003995], (pKP13c), [GenBank: CP003997] (pKP13d), [GenBank: CP003998] (pKP13e) and [GenBank: CP004000] (pKP13f).

## Abbreviations

CDS: Coding sequence; KPC: *Klebsiella pneumoniae* carbapenemase; MDR: Multi-drug resistant; MIC: Minimum inhibitory concentration; RGP: Region of genomic plasticity; SNP: Single-nucleotide polymorphism.

## Competing interests

The authors declare that there are no competing interests.

## Authors’ contributions

ECV and MP provided the Kp13 isolate and performed bacterial identification. ATRV and MFN conceived the pyrosequencing strategy. Manual annotation and bioinformatic analyses were performed by ACG, ACPV, CM, DEX, FGB, LGPA, MFN, NCBL, PIPR, RCP and RG. SNPs analysis was performed by NCBL and MFN. The maintenance and update of KlebsiellaScope in the MicroScope platform was performed by CM and her team. The manuscript was prepared by PIPR, MFN, RCP and ACG. All authors read and approved the final manuscript.

## Supplementary Material

Additional file 1 **Chromosomal architecture of the four compared *K. pneumoniae *** **isolates.** Multiple alignments among the chromosomes of Kp13, 342, NTUH-K2044 and MGH 78578 performed using the Mauve software. Each line represents the linearized chromosome of the compared strain. Rectangles in different colors represent locally colinear blocks (LCBs) and homologous LCBs among strains are connected by vertical lines. The white portions within LCBs do not exhibit correspondence in the compared bacterial strains. Click here for file

Additional file 2 **Distribution of locally colinear blocks (LCBs) of *K. pneumoniae *** **Kp13 relative to the NTUH-K2044, MGH 78578 and 342 strains.** Click here for file

Additional file 3 **Regions of genomic plasticity.** Detailed analysis of each RGP detected in the Kp13 chromosome. Click here for file

Additional file 4 **Shared and conserved genes between *K. pneumoniae *** **strains 342, MGH 78578, NTUH-K2044 and Kp13.** The roman number labeling each spreadsheet corresponds to the partial intersections indicated in Figure 4. Click here for file

Additional file 5 **Iron scavenging systems in compared *K. pneumoniae *** **strains.** Click here for file

Additional file 6 **Adhesins detected in compared *K. pneumoniae *** **strains.** Click here for file

Additional file 7 Selected common SNPs falling within virulence- and resistance-related CDSs identified in comparisons with the strains NTUH-K2044 and MGH 78578. Click here for file

Additional file 8 **Multiple alignment of the region upstream *ompK35 *** **in *K. pneumoniae *** **strains Kp13 and NVT2001 and outer membrane proteins of Kp13.***Panel A*, The alignment was performed relative to *K. pneumoniae* NVT2001, studied in [60]. The marked regions in the sequence of NVT2001 correspond to those transferred by similarity from *ompF* in *E. coli* by those authors, and include IHF (integration host factor), RBS (ribosome binding site), the −10 and −35 promoters and the OmpR binding sites (F1-F4) detected in NVT2001. Due to the transposase recombination event that took place in Kp13, there are several differences from the comparison with strain NVT2001 that should affect *ompK35* expression in Kp13. *Panel B,* Sodium dodecyl sulfate-polyacrylamide gel electrophoretic analysis of outer membrane proteins (OMPs) from Kp13 strain. Lanes 1 and 6, molecular marker weight; lane 2, *K. pneumoniae* control strain 194 exhibiting intact OmpK35 and OmpK36 porins; lanes 3–5, OMP profiles of Kp13 strain. Click here for file

Additional file 9 Total number of common SNPs falling within intergenic regions and CDSs grouped by COG classification identified in comparisons with the strains NTUH-K2044 and MGH 78578. Click here for file

## References

[B1] Podschun R Ullmann U *Klebsiella* spp. as nosocomial pathogens: epidemiology, taxonomy, typing methods, and pathogenicity factors Clin Microbiol Rev 1998 15 589 603 9767057 10.1128/cmr.11.4.589 PMC88898

[B2] Podschun R Pietsch S Holler C Ullmann U Incidence of *Klebsiella* species in surface waters and their expression of virulence factors Appl Environ Microbiol 2001 15 3325 3327 10.1128/AEM.67.7.3325-3327.2001 11425763 PMC93022

[B3] Wu K-M Li L-H Yan J-J Tsao N Liao T-L Tsai H-C Fung C-P Chen H-J Liu Y-M Wang J-T Fang C-T Chang S-C Shu H-Y Liu T-T Chen Y-T Shiau Y-R Lauderdale T-L Su I-J Kirby R Tsai S-F Genome sequencing and comparative analysis of *Klebsiella pneumoniae* NTUH-K2044, a strain causing liver abscess and meningitis J Bacteriol 2009 15 4492 4501 10.1128/JB.00315-09 19447910 PMC2704730

[B4] Fouts DE Tyler HL DeBoy RT Daugherty S Ren Q Badger JH Durkin AS Huot H Shrivastava S Kothari S Dodson RJ Mohamoud Y Khouri H Roesch LFW Krogfelt KA Struve C Triplett EW Methé BA Complete genome sequence of the N2-fixing broad host range endophyte *Klebsiella pneumoniae* 342 and virulence predictions verified in mice PLoS Genet 2008 15 e1000141 10.1371/journal.pgen.1000141 18654632 PMC2453333

[B5] Ogawa W Li D-W Yu P Begum A Mizushima T Kuroda T Tsuchiya T Multidrug resistance in *Klebsiella pneumoniae* MGH78578 and cloning of genes responsible for the resistance Biological and Pharmaceutical Bulletin 2005 15 1505 1508 10.1248/bpb.28.1505 16079502

[B6] Liu P Li P Jiang X Bi D Xie Y Tai C Deng Z Rajakumar K Ou H-Y Complete genome sequence of *Klebsiella pneumoniae* subsp. *pneumoniae* HS11286, a multidrug-resistant strain isolated from human sputum J Bacteriol 2012 15 1841 1842 10.1128/JB.00043-12 22408243 PMC3302456

[B7] Shin SH Kim S Kim JY Lee S Um Y Oh M-K Kim Y-R Lee J Yang K-S Complete genome sequence of the 2,3-butanediol-producing *Klebsiella pneumoniae* strain KCTC 2242 J Bacteriol 2012 15 2736 2737 10.1128/JB.00027-12 22535926 PMC3347200

[B8] Lin A-C Liao T-L Lin Y-C Lai Y-C Lu M-C Chen Y-T Complete genome sequence of *Klebsiella pneumoniae* 1084, a hypermucoviscosity-negative K1 clinical strain J Bacteriol 2012 15 6316 10.1128/JB.01548-12 23105059 PMC3486373

[B9] Livermore DM Woodford N The β-lactamase threat in *Enterobacteriaceae*, *Pseudomonas* and *Acinetobacter* Trends Microbiol 2006 15 413 420 10.1016/j.tim.2006.07.008 16876996

[B10] Seki LM Pereira PS Seki LM Pereira PS de Souza Mda P Conceição MS Marques EA Porto CO Colnago EML Alves CFM Gomes D Carvalho-Assef APD Samuelsen Ø Asensi MD Molecular epidemiology of KPC-2- producing Klebsiella pneumoniae isolates in Brazil: the predominance of sequence type 437 Diagn Microbiol Infect Dis 2011 15 274 277 10.1016/j.diagmicrobio.2011.01.006 21397425

[B11] Pereira PS de Araujo CFM Seki LM Zahner V Carvalho-Assef APD Asensi MD Update of the molecular epidemiology of KPC-2-producing *Klebsiella pneumoniae* in Brazil: spread of clonal complex 11 (ST11, ST437 and ST340) J Antimicrob Chemother 2013 15 312 316 10.1093/jac/dks396 23070735

[B12] Ramos PI Picão RC Vespero EC Pelisson M Zuleta LFG Almeida LGP Gerber AL Vasconcelos ATR Gales AC Nicolás MF Pyrosequencing-based analysis reveals a novel capsular gene cluster in a KPC-producing *Klebsiella pneumoniae* clinical isolate identified in Brazil BMC Microbiol 2012 15 173 10.1186/1471-2180-12-173 22882772 PMC3438125

[B13] Darling AE Mau B Blattner FR Perna NT Mauve: multiple alignment of conserved genomic sequence with rearrangements Genome Res 2004 15 1394 1403 10.1101/gr.2289704 15231754 PMC442156

[B14] Hacker J Carniel E Ecological fitness, genomic islands and bacterial pathogenicity. A Darwinian view of the evolution of microbes EMBO Rep 2001 15 376 381 10.1093/embo-reports/kve097 11375927 PMC1083891

[B15] Kittichotirat W Bumgarner R Chen C Markedly different genome arrangements between serotype a strains and serotypes b or c strains of *Aggregatibacter actinomycetemcomitans* BMC Genomics 2010 15 489 10.1186/1471-2164-11-489 20825670 PMC2996985

[B16] Ochman H Lawrence JG Groisman EA Lateral gene transfer and the nature of bacterial innovation Nature 2000 15 299 304 10.1038/35012500 10830951

[B17] Pieper DH Santos VAP M d Golyshin PN Genomic and mechanistic insights into the biodegradation of organic pollutants Curr Opin Biotechnol 2004 15 215 224 10.1016/j.copbio.2004.03.008 15193329

[B18] Casjens SR Gilcrease EB Huang WM Bunny KL Pedulla ML Ford ME Houtz JM Hatfull GF Hendrix RW The pKO2 linear plasmid prophage of *Klebsiella oxytoca* J Bacteriol 2004 15 1818 1832 10.1128/JB.186.6.1818-1832.2004 14996813 PMC355964

[B19] Bönemann G Pietrosiuk A Mogk A Tubules and donuts: a type VI secretion story Mol Microbiol 2010 15 815 821 10.1111/j.1365-2958.2010.07171.x 20444095

[B20] Mougous JD Cuff ME Raunser S Shen A Zhou M Gifford CA Goodman AL Joachimiak G Ordoñez CL Lory S Walz T Joachimiak A Mekalanos JJ A virulence locus of *Pseudomonas aeruginosa* encodes a protein secretion apparatus Science 2006 15 1526 1530 10.1126/science.1128393 16763151 PMC2800167

[B21] Cascales E Cambillau C Structural biology of type VI secretion systems Phil Trans Roy Soc Lond 2012 15 1102 1111 10.1098/rstb.2011.0209 22411981 PMC3297440

[B22] Bingle LE Bailey CM Pallen MJ Type VI secretion: a beginner’s guide Curr Opin Microbiol 2008 15 3 8 10.1016/j.mib.2008.01.006 18289922

[B23] Suarez G Sierra JC Kirtley ML Chopra AK Role of Hcp, a type 6 secretion system effector, of *Aeromonas hydrophila* in modulating activation of host immune cells Microbiology 2010 15 Pt 12 3678 3688 20798163 10.1099/mic.0.041277-0 PMC3068704

[B24] Lin T Lee C-Z Hsieh P-F Tsai S Wang J Characterization of integrative and conjugative element ICE*Kp1*-associated genomic heterogeneity in a *Klebsiella pneumoniae* strain isolated from a primary liver abscess J Bacteriol 2008 15 515 526 10.1128/JB.01219-07 17981959 PMC2223707

[B25] Ogawa W Onishi M Ni R Tsuchiya T Kuroda T Functional study of the novel multidrug efflux pump KexD from *Klebsiella pneumoniae* Gene 2012 15 177 182 10.1016/j.gene.2012.02.008 22391093

[B26] Deng WL Chang HY Peng HL Pengi H Acetoin catabolic system of *Klebsiella pneumoniae* CG43: sequence, expression, and organization of the *aco* operon J Bacteriol 1994 15 3527 3535 8206829 10.1128/jb.176.12.3527-3535.1994 PMC205540

[B27] Brilli M Liò P Lacroix V Sagot M-F Short and long-term genome stability analysis of prokaryotic genomes BMC Genomics 2013 15 309 10.1186/1471-2164-14-309 23651581 PMC3683328

[B28] Imazu K Tanaka S Kuroda A Anbe Y Kato J Ohtake H Enhanced utilization of phosphonate and phosphite by *Klebsiella aerogenes* Appl Environ Microbiol 1998 15 3754 3758 9758795 10.1128/aem.64.10.3754-3758.1998 PMC106539

[B29] Wang W Xi H Bi Q Hu Y Zhang Y Ni M Cloning, expression and characterization of d-aminoacylase from *Achromobacter xylosoxidans* subsp. *denitrificans* ATCC 15173 Microbiol Res 2013 15 360 366 10.1016/j.micres.2013.01.002 23369306

[B30] Wiame E Duquenne A Delpierre G Van Schaftingen E Identification of enzymes acting on a-glycated amino acids in *Bacillus subtilis* FEBS Lett 2004 15 469 472 10.1016/j.febslet.2004.10.049 15556630

[B31] Friedman EA Advanced glycosylated end products and hyperglycemia in the pathogenesis of diabetic complications Diabetes Care 1999 15 B65 B71 10.2337/diacare.22.1.65 10097902

[B32] Schaible UE Kaufmann SHE Iron and microbial infection Nat Rev 2004 15 946 953 10.1038/nrmicro1046 15550940

[B33] Hsieh P-F Lin T-L Lee C-Z Tsai S-F Wang J-T Serum-induced iron-acquisition systems and TonB contribute to virulence in *Klebsiella pneumoniae* causing primary pyogenic liver abscess J Infect Dis 2008 15 1717 1727 10.1086/588383 18433330

[B34] Russo TA Shon AS Beanan JM Olson R MacDonald U Pomakov AO Visitacion MP Hypervirulent *K. pneumoniae* secretes more and more active iron-acquisition molecules than “Classical” *K. pneumoniae* thereby enhancing its Virulence PLoS ONE 2011 15 e26734 10.1371/journal.pone.0026734 22039542 PMC3200348

[B35] Garénaux A Caza M Dozois CM The Ins and Outs of siderophore mediated iron uptake by extra-intestinal pathogenic *Escherichia coli* Vet Microbiol 2011 15 89 98 10.1016/j.vetmic.2011.05.023 21680117

[B36] Wilson JW Schurr MJ LeBlanc CL Ramamurthy R Buchanan KL Nickerson CA Mechanisms of bacterial pathogenicity Postgrad Med J 2002 15 216 224 10.1136/pmj.78.918.216 11930024 PMC1742320

[B37] Struve C Bojer M Krogfelt KA Characterization of *Klebsiella pneumoniae* type 1 fimbriae by detection of phase variation during colonization and infection and impact on virulence Infect Immun 2008 15 4055 4065 10.1128/IAI.00494-08 18559432 PMC2519443

[B38] Ong C-LY Beatson SA Totsika M Forestier C McEwan AG Schembri MA Molecular analysis of type 3 fimbrial genes from *Escherichia coli.* Klebsiella and Citrobacter species BMC Microbiol 2010 15 183 10.1186/1471-2180-10-183 20576143 PMC2900259

[B39] Wu C-C Huang Y-J Fung C-P Peng H-L Regulation of the *Klebsiella pneumoniae* Kpc fimbriae by the site-specific recombinase KpcI Microbiology 2010 15 1983 1992 10.1099/mic.0.038158-0 20378654

[B40] Wizemann TM Adamou JE Langermann S Adhesins as targets for vaccine development Emerg Infect Dis 1999 15 395 403 10.3201/eid0503.990310 10341176 PMC2640765

[B41] Haeggman S Löfdahl S Paauw A Verhoef J Brisse S Diversity and evolution of the class A chromosomal beta-lactamase gene in *Klebsiella pneumoniae* Antimicrob Agents Chemother 2004 15 2400 2408 10.1128/AAC.48.7.2400-2408.2004 15215087 PMC434173

[B42] Bradford PA Extended-spectrum beta-lactamases in the 21st century: characterization, epidemiology, and detection of this important resistance threat Clin Microbiol Rev 2001 15 933 951 10.1128/CMR.14.4.933-951.2001 11585791 PMC89009

[B43] Poirel L Naas T Nordmann P Diversity, epidemiology, and genetics of class D beta-lactamases Antimicrob Agents Chemother 2010 15 24 38 10.1128/AAC.01512-08 19721065 PMC2798486

[B44] D’Andrea MM Arena F Pallecchi L Rossolini GM CTX-M-type β-lactamases: A successful story of antibiotic resistance Int J Med Microbiol 2013 15 305 317 10.1016/j.ijmm.2013.02.008 23490927

[B45] Arduino SM Roy PH Jacoby GA Orman BE Pineiro SA Centron D *bla*_CTX-M-2_ Is Located in an Unusual Class 1 Integron (In*35*) Which Includes Orf513 Antimicrob Agents Chemother 2002 15 2303 2306 10.1128/AAC.46.7.2303-2306.2002 12069995 PMC127297

[B46] Clímaco EC Minarini LAR Lúcia A CTX-M-producing *Klebsiella* spp. in a Brazilian hospital: what has changed in 6 years? Diagn Microbiol Infect Dis 2010 15 186 189 10.1016/j.diagmicrobio.2010.05.013 20846594

[B47] Poirel L Le Thomas I Naas T Karim A Nordmann P Biochemical sequence analyses of GES-1, a novel class A extended-spectrum beta-lactamase, and the class 1 integron In*52* from *Klebsiella pneumoniae* Antimicrob Agents Chemother 2000 15 622 632 10.1128/AAC.44.3.622-632.2000 10681329 PMC89737

[B48] Naas T Cuzon G Villegas M Lartigue M-F Quinn JP Nordmann P Genetic structures at the origin of acquisition of the beta-lactamase *bla*_KPC_ gene Antimicrob Agents Chemother 2008 15 1257 1263 10.1128/AAC.01451-07 18227185 PMC2292522

[B49] Naville M Ghuillot-Gaudeffroy A Marchais A Gautheret D ARNold: a web tool for the prediction of Rho-independent transcription terminators RNA Biol 2011 15 11 13 10.4161/rna.8.1.13346 21282983

[B50] Fu Y Guo L Xu Y Zhang W Gu J Xu J Chen X Zhao Y Ma J Liu X Zhang F Alteration of GyrA amino acid required for ciprofloxacin resistance in *Klebsiella pneumoniae* isolates in China Antimicrob Agents Chemother 2008 15 2980 2983 10.1128/AAC.00151-08 18505849 PMC2493132

[B51] Deguchi T Fukuoka A Yasuda M Nakano M Ozeki S Kanematsu E Nishino Y Ishihara S Ban Y Kawada Y Alterations in the GyrA subunit of DNA gyrase and the ParC subunit of topoisomerase IV in quinolone-resistant clinical isolates of *Klebsiella pneumoniae* Antimicrob Agents Chemother 1997 15 699 701 9056017 10.1128/aac.41.3.699 PMC163775

[B52] Wachino J Arakawa Y Exogenously acquired 16S rRNA methyltransferases found in aminoglycoside-resistant pathogenic Gram-negative bacteria: an update Drug Resist Updat 2012 15 133 148 10.1016/j.drup.2012.05.001 22673098

[B53] Bueno MFC Francisco GR O’Hara JA de Oliveira Garcia D Doi Y Coproduction of 16S rRNA methyltransferase RmtD or RmtG with KPC-2 and CTX-M group extended-spectrum β-lactamases in *Klebsiella pneumoniae* Antimicrob Agents Chemother 2013 15 2397 2400 10.1128/AAC.02108-12 23459483 PMC3632927

[B54] Veiga DFT Vicente FFR Nicolás MF Vasconcelos ATR Predicting transcriptional regulatory interactions with artificial neural networks applied to *E. coli* multidrug resistance efflux pumps BMC Microbiol 2008 15 101 114 10.1186/1471-2180-8-101 18565227 PMC2453137

[B55] Bialek-Davenet S Marcon E Leflon-Guibout V Lavigne J-P Bert F Moreau R Nicolas-Chanoine M-H In vitro selection of *ramR* and *soxR* mutants overexpressing efflux systems by fluoroquinolones as well as cefoxitin in *Klebsiella pneumoniae* Antimicrob Agents Chemother 2011 15 2795 2802 10.1128/AAC.00156-11 21464248 PMC3101381

[B56] Perez F Rudin SD Marshal SH Coakley P Chen L Kreiswirth BN Rather PN Hujer AM Toltzis P van Duin D Paterson DL Bonomo RA OqxAB, a Quinolone and Olaquindox Efflux Pump, is Widely Distributed among Multidrug Resistant *Klebsiella pneumoniae* of Human Origin Antimicrob Agents Chemother 2013 15 4602 4603 10.1128/AAC.00725-13 23817374 PMC3754307

[B57] Hansen LH Jensen LB Sørensen HI Sørensen SJ Substrate specificity of the OqxAB multidrug resistance pump in *Escherichia coli* and selected enteric bacteria J Antimicrob Chemother 2007 15 145 147 10.1093/jac/dkm167 17526501

[B58] Nishino K Yamaguchi A Analysis of a complete library of putative drug transporter genes in *Escherichia coli* J Bacteriol 2001 15 5803 5812 10.1128/JB.183.20.5803-5812.2001 11566977 PMC99656

[B59] Hernández-Allés S Albertí S Alvarez D Doménech-Sánchez A Martínez-Martínez L Gil J Tomás JM Benedí VJ Porin expression in clinical isolates of *Klebsiella pneumoniae* Microbiology 1999 15 673 679 10.1099/13500872-145-3-673 10217501

[B60] Tsai Y-K Fung C-P Lin J-C Chen J-H Chang F-Y Chen T-L Siu LK *Klebsiella pneumoniae* outer membrane porins OmpK35 and OmpK36 play roles in both antimicrobial resistance and virulence Antimicrob Agents Chemother 2011 15 1485 1493 10.1128/AAC.01275-10 21282452 PMC3067157

[B61] Llobet E Campos MA Giménez P Moranta D Bengoechea JA Analysis of the networks controlling the antimicrobial-peptide-dependent induction of *Klebsiella pneumoniae* virulence factors Infect Immun 2011 15 3718 3732 10.1128/IAI.05226-11 21708987 PMC3165464

[B62] Martínez J Martínez L Rosenblueth M Silva J Martínez-Romero E How are gene sequence analyses modifying bacterial taxonomy? The case of *Klebsiella* Int Microbiol 2004 15 261 268 15666246

[B63] Clinical and Laboratory Standards Institute M02-A11, Approved Standard, Performance Standards for Antimicrobial Disk Susceptibility Tests 2012 Wayne, PA: CLSI

[B64] Clinical and Laboratory Standards Institute Performance Standards for Antimicrobial Susceptibility Testing. 23rd Informational Supplement 2013 Wayne, PA: CLSI

[B65] Wyeth Pharmaceuticals Tygacil [package insert] 2005 Philadelphia, PA: Wyeth Pharmaceuticals Inc

[B66] European Committee on Antimicrobial Susceptibility Testing Breakpoint Tables for Interpretation of MICs and Zone Diameters, Version 3.0 2013 EUCAST: Basel, Switzerland

[B67] Almeida LGP Paixão R Souza RC Costa GC Barrientos FJA dos Santos MT Almeida DF Vasconcelos ATR A System for Automated Bacterial (genome) Integrated Annotation–SABIA Bioinformatics 2004 15 2832 2833 10.1093/bioinformatics/bth273 15087310

[B68] Diancourt L Passet V Verhoef J Grimont PAD Brisse S Multilocus sequence typing of *Klebsiella pneumoniae* nosocomial isolates J Clin Microbiol 2005 15 4178 4182 10.1128/JCM.43.8.4178-4182.2005 16081970 PMC1233940

[B69] Francisco AP Vaz C Monteiro PT Melo-Cristino J Ramirez M Carriço JA PHYLOViZ: phylogenetic inference and data visualization for sequence based typing methods BMC Bioinformatics 2012 15 87 10.1186/1471-2105-13-87 22568821 PMC3403920

[B70] Vallenet D Engelen S Mornico D Cruveiller S Fleury L Lajus A Rouy Z Roche D Salvignol G Scarpelli C Médigue C MicroScope: a platform for microbial genome annotation and comparative genomics Database 2009 15 bap021 20157493 10.1093/database/bap021 PMC2790312

[B71] Vallenet D Belda E Calteau A Cruveiller S Engelen S Lajus A Le Fèvre F Longin C Mornico D Roche D Rouy Z Salvignol G Scarpelli C Thil Smith AA Weiman M Médigue C MicroScope–an integrated microbial resource for the curation and comparative analysis of genomic and metabolic data Nucleic Acids Res 2013 15 Database issue D636 D647 23193269 10.1093/nar/gks1194 PMC3531135

[B72] Vernikos GS Parkhill J Interpolated variable order motifs for identification of horizontally acquired DNA: revisiting the *Salmonella* pathogenicity islands Bioinformatics 2006 15 2196 2203 10.1093/bioinformatics/btl369 16837528

[B73] Waack S Keller O Asper R Brodag T Damm C Fricke WF Surovcik K Meinicke P Merkl R Score-based prediction of genomic islands in prokaryotic genomes using hidden Markov models BMC Bioinformatics 2006 15 142 10.1186/1471-2105-7-142 16542435 PMC1489950

[B74] Sullivan MJ Petty NK Beatson SA Easyfig: a genome comparison visualizer Bioinformatics 2011 15 1009 1010 10.1093/bioinformatics/btr039 21278367 PMC3065679

[B75] Alikhan N-F Petty NK Ben Zakour NL Beatson SA BLAST Ring Image Generator (BRIG): simple prokaryote genome comparisons BMC Genomics 2011 15 402 10.1186/1471-2164-12-402 21824423 PMC3163573

[B76] Janssen PJ Van Houdt R Moors H Monsieurs P Morin N Michaux A Benotmane MA Leys N Vallaeys T Lapidus A Monchy S Médigue C Taghavi S McCorkle S Dunn J van der Lelie D Mergeay M The complete genome sequence of *Cupriavidus metallidurans* strain CH34, a master survivalist in harsh and anthropogenic environments PLoS ONE 2010 15 e10433 10.1371/journal.pone.0010433 20463976 PMC2864759

[B77] Chen H Boutros PC VennDiagram: a package for the generation of highly-customizable Venn and Euler diagrams in R BMC Bioinformatics 2011 15 35 10.1186/1471-2105-12-35 21269502 PMC3041657

[B78] Darling AE Mau B Perna NT progressiveMauve: multiple genome alignment with gene gain, loss and rearrangement PLoS ONE 2010 15 e11147 10.1371/journal.pone.0011147 20593022 PMC2892488

[B79] Zhou Y Liang Y Lynch KH Dennis JJ Wishart DS PHAST: a fast phage search tool Nucleic Acids Res 2011 15 Web Server issue W347 W352 21672955 10.1093/nar/gkr485 PMC3125810

[B80] Quispe Saji del R G Costa MOC Netto DS Lima NCB Klein CC Vasconcelos ATR Nicolás MF AtlasT4SS: a curated database for type IV secretion systems BMC Microbiol 2012 15 172 10.1186/1471-2180-12-172 22876890 PMC3489848

[B81] Liu B Pop M ARDB–Antibiotic Resistance Genes Database Nucleic Acids Res 2009 15 Database issue D443 D447 18832362 10.1093/nar/gkn656 PMC2686595

[B82] Siguier P Perochon J Lestrade L Mahillon J Chandler M ISfinder: the reference centre for bacterial insertion sequences Nucleic Acids Res 2006 15 Database issue D32 D36 16381877 10.1093/nar/gkj014 PMC1347377

[B83] Lima NCB Computational Approach for Detection and Analysis of Single-Nucleotide Polymorphisms (SNPs) in Bacterial Genomes MsC thesis 2011 Petrópolis, Brazil: National Laboratory for Scientific Computing

[B84] Kurtz S Phillippy A Delcher AL Smoot M Shumway M Antonescu C Salzberg SL Versatile and open software for comparing large genomes Genome Biol 2004 15 R12 10.1186/gb-2004-5-2-r12 14759262 PMC395750

