# Supplementary figures and images for: Comparative analysis of the complete genome of KPC-2-producing Klebsiella pneumoniae Kp13 reveals remarkable genome plasticity and a wide repertoire of virulence and resistance mechanisms
Source: BMC Genomics. 2014 Jan 22;15:54. doi: 10.1186/1471-2164-15-54 (PMC3904158; doi:10.1186/1471-2164-15-54)

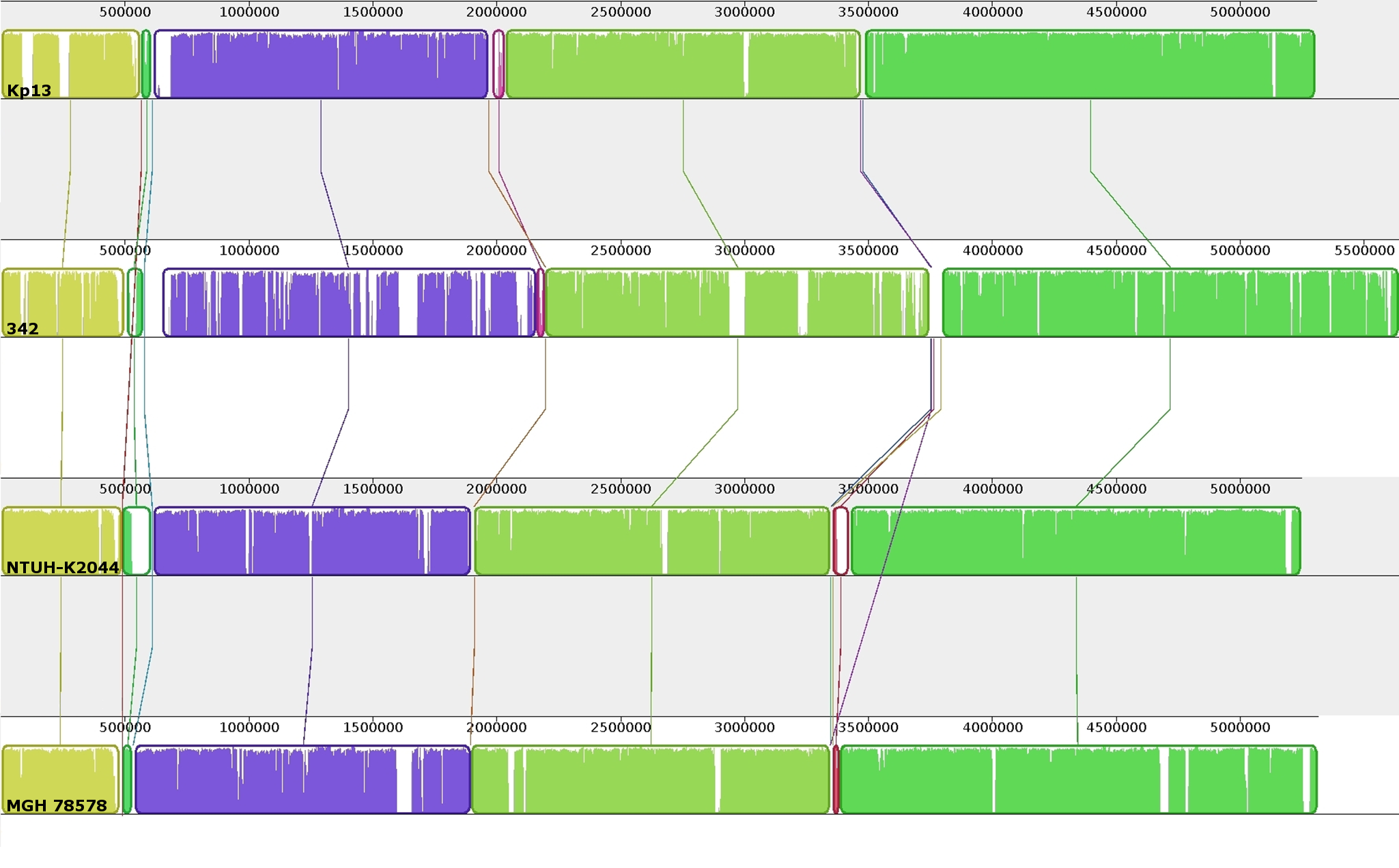

Supplement: Additional file 1 — Chromosomal architecture of the four compared K. pneumoniae isolates. Multiple alignments among the chromosomes of Kp13, 342, NTUH-K2044 and MGH 78578 performed using the Mauve software. Each line represents the linearized chromosome of the compared strain. Rectangles in different colors represent locally colinear blocks (LCBs) and homologous LCBs among strains are connected by vertical lines. The white portions within LCBs do not exhibit correspondence in the compared bacterial strains. [file 1471-2164-15-54-S1.tiff]

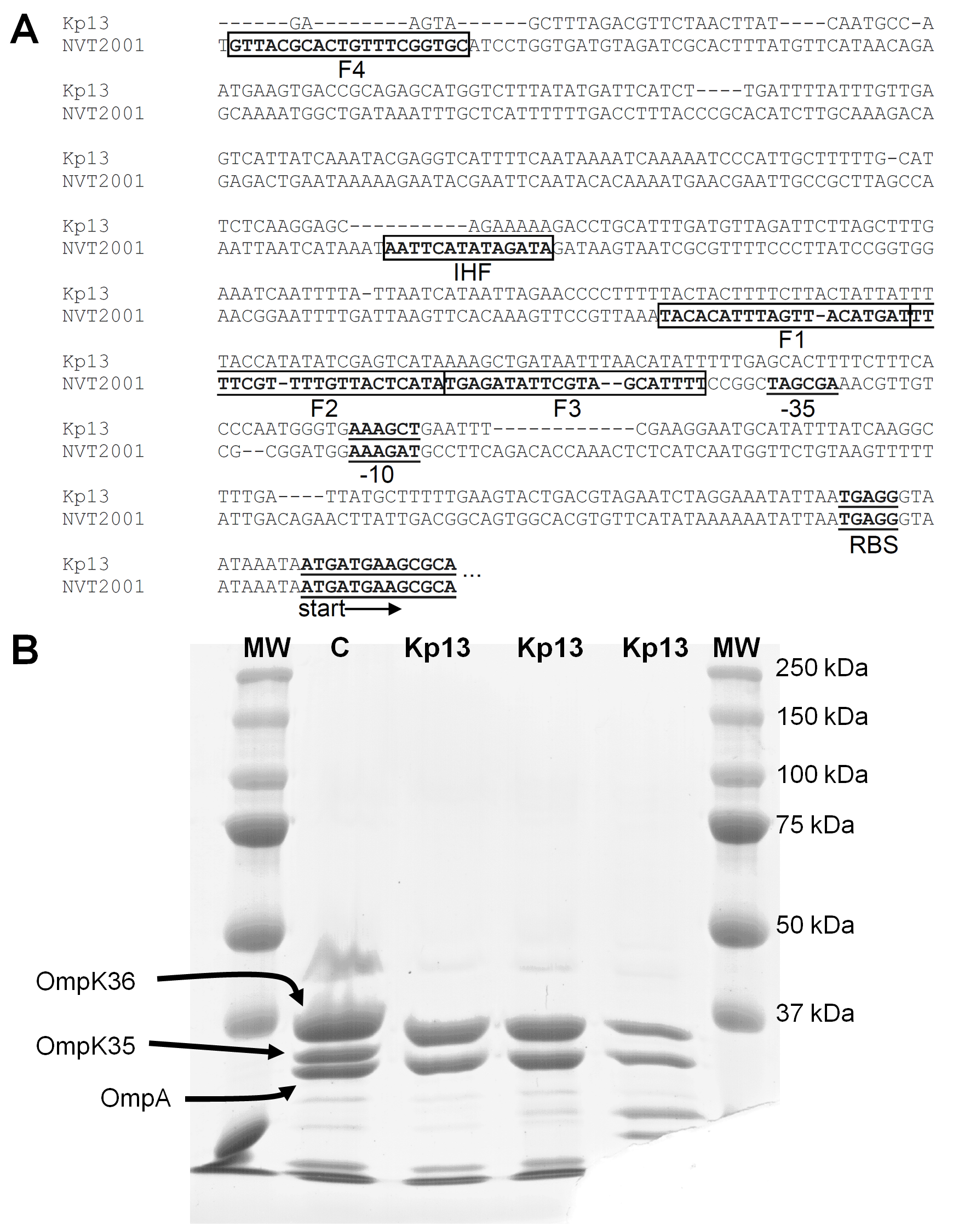

Supplement: Additional file 8 — Multiple alignment of the region upstream ompK35 in K. pneumoniae strains Kp13 and NVT2001 and outer membrane proteins of Kp13.Panel A, The alignment was performed relative to K. pneumoniae NVT2001, studied in [60]. The marked regions in the sequence of NVT2001 correspond to those transferred by similarity from ompF in E. coli by those authors, and include IHF (integration host factor), RBS (ribosome binding site), the −10 and −35 promoters and the OmpR binding sites (F1-F4) detected in NVT2001. Due to the transposase recombination event that took place in Kp13, there are several differences from the comparison with strain NVT2001 that should affect ompK35 expression in Kp13. Panel B, Sodium dodecyl sulfate-polyacrylamide gel electrophoretic analysis of outer membrane proteins (OMPs) from Kp13 strain. Lanes 1 and 6, molecular marker weight; lane 2, K. pneumoniae control strain 194 exhibiting intact OmpK35 and OmpK36 porins; lanes 3–5, OMP profiles of Kp13 strain. [file 1471-2164-15-54-S8.tiff]
