# Supplementary material for: Comparative analysis of the complete genome of KPC-2-producing Klebsiella pneumoniae Kp13 reveals remarkable genome plasticity and a wide repertoire of virulence and resistance mechanisms
Source: BMC Genomics. 2014 Jan 22;15:54. doi: 10.1186/1471-2164-15-54 (PMC3904158; doi:10.1186/1471-2164-15-54)
Supplement: Additional file 3 — Regions of genomic plasticity. Detailed analysis of each RGP detected in the Kp13 chromosome. [file 1471-2164-15-54-S3.pdf]

Additional file 8 - Regions of genomic plasticity

RGP1

This region is also present in the chromosome of strain MGH 78578, but not in those of strains 342 and NTUH-K2044. An insertion sequence (KP05176) flanks the 5' terminal of this RGP and could have mediated its integration. This region is 15.4 kbp in length and contains 17 predicted CDSs (Table 2). *In silico* domain analyses revealed the presence of multiple domains within the CDSs that form this RGP including aldehyde dehydrogenase (KP02706, InterPro accession no. IPR015590), aspartate dehydrogenase (KP31638, InterPro accession no. IPR002811) and two Rieske-type domains which are common to many non-haem iron oxygenases. The latter may be involved in aromatic compounds degradation [1]. Possible transporters belonging to the major facilitator superfamily (KP02711 and KP02721) are also present, and an CDS with a possible role in regulation (KP02714) was identified. The protein sequence of this CDS contains at both termini two HTH IclR domains (InterPro accession nos. IPR005471 and IPR014757) which participate in the regulation of a variety of metabolic processes including degradation of aromatics [2]. An interesting pattern in this region is the localization of the predicted CDSs on the same DNA strand (except for KP02721) as well as the relatively small intergenic distances between them, the greatest being of 90 bp between KP02713 and KP02714, which might be indicative that the expression of the genes in this RGP might be coupled and that they may have a role on a functionally related process.

This region is not found in *K. pneumoniae* NTUH-K2044, although there are evidences that it could have been lost during the evolutionary course of this genome. In NTUH-K2044, the CDS KP1_4647 is located adjacent to *speB*, the conserved gene that flanks the 5' terminal of this region in all strains compared. KP1_4647 is composed of 153 nt and was annotated as a possible membrane protein with the best hits in MGH 78578 and Kp13 being, respectively, KPN_03359 and KP02704, both having 1,269 nt in size. We hypothesize that this region suffered deletion in NTUH-K2044 and that this event led to the truncation of this CDS which resulted in its smaller size relative to the other studied *K. pneumoniae*. On the other hand, in the chromosome of strain 342 (where this RGP is also absent) the gene for this membrane protein is found intact and is 1,269 in length (KPK_0739).

RGP2

This plasticity segment contains transposases (KP02533, KP02534), a phage integrase (KP16268) as well as CDSs annotated as hypothetical. The conserved genes that flank this region at its 5' and 3' termini are, respectively, *mutS* and *hmuV*. In strain 342 and NTUH-K2044 they are contiguously localized and this region represents an unique insertion within the Kp13 chromosome. In the chromosome of MGH 78578 these genes are not found adjacent and there exists two CDSs between them, which were annotated as an aminoacyl-histidine dipeptidase (locus ID KPN_03092) and tagaturonate reductase (KPN_03093).

RGP3 and RGP9 (bacteriophage-related)

These RGPs represent probable phage insertions in the Kp13 chromosome. In RGP3, identical *att* sites were detected flanking the termini of the region (*attL*/*attR*,

ATTGAGTGGGAAT) where recombination between phage and bacterial DNA probably occurred. The *guaA* gene (locus KP00870 in Kp13) is conserved among the compared strains and flanks the 5' region of this RGP, and it has been previously reported that the attachment site that follows this gene functions as recombination spot between *Salmonella* species and ε15 phages [3] and the same mechanism might have occured in *K. pneumoniae* Kp13 and related strains. There are no insertions in this region in the chromosomes of strains NTUH-K2044 and MGH 78578, while the analog region in *K. pneumoniae* 342 harbors a distinct 12.9 kbp insertion that may represent an integrated plasmid carrying genes related to plasmidial replication and mobilization [4]. The CDSs predicted in this region code for many hypothetical proteins as well as methylases (KP00873, KP00885) which could have a role in protecting the inserted DNA against the action of bacterial restriction enzymes. The phage genes identified in RGP3 using the PHAST webserver code for multiple bacteriophage-related functions, including tail and capsid formation, fibers, proteases and terminases.

The second bacteriophage-related RGP was designated RGP9. At its initial portion a variety of CDSs unrelated to phage metabolism are found, and their predicted domains reveal a possible signal transduction role (KP04084) as well as enzymatic activities such as oxidoreductase (KP04069), phosphohydrolase (KP04085) and asparaginase (KP04086). Also in this region are two CDSs displaying high amino acid identity to CioA and CioB from *Pseudomonas aeruginosa*, KP04075 (70.95% BLASTP identity to UniProtKB accession O07440) and KP04076 (66.07% identity to accession O07441). In *P. aeruginosa*, these proteins are cyanide-insensitive terminal oxidases which could provide a way to sustain aerobic respiration in the presence of cyanogenic compounds [5]. If these proteins are indeed expressed by Kp13 and related *K. pneumoniae* it would

offer a mechanism to compete against cyanide-producing organisms. The central and 3' terminal part of RGP9 contain phage-related genes and their insertion occurs next to the conserved *icd* gene (KP04861) which lies outside the boundaries of this plasticity segment. Previous reports indicate that the *icd* gene in *Escherichia coli* harbors an integration spot at its 3' terminal which facilitates prophage insertion through homologous recombination [6,7]. Most CDSs in this region display similarity to the ones found in plasmidial prophage *ϕKO2* found in *K. oxytoca.* However, the coverage of the Kp13 region to this prophage is limited to the segments responsible for bacteriophage head and tail formation, as the initial portion of *ϕKO2* contains genes involved in plasmid mobilization. In strains 342 and NTUH-K2044 non-identical phage insertions also took place, and while the regions present in 342 and Kp13 share limited similarity, the inserted elements at each one of them have distinct organization and are probably the result of independent recombination events. The NTUH-K2044 region also lacks the initial segment that is found in Kp13, 342 and MGH 78578 containing CDSs unrelated to phage metabolism.

RGP4, RGP6 and RGP8 (T6SS-related)

The DNA segment comprised within RGP4 is the first of three identified T6SS in the Kp13 chromosome. RGP4 is flanked in its 3' terminal by an Arg-tRNA (located between coordinates 1,481,583-1,481,657) and it contains multiple CDSs with predicted T6SS-related domains, such as IcmF (KP01067) and DotU (IcmH, KP03057). Although both proteins have been initially classified as belonging to the T4SS, being homologous to the Icm/Dot archetype of *Legionella pneumophila*, they are both equally found in t6ss [8,9]. Only two (ClpV and FHA domain-containing protein [COG3456]) from a total of 14 proteins described by Bingle *et al.* [9] as the most conserved in this secretion system

could not be identified in this region. ClpV is the energizing component of this system, while FHA-domain homologs regulate protein secretion via phosphorylation of its threonine residues [10]. However, a ClpV homolog (KP04342) was identified at a distinct T6SS region in Kp13 and could possibly be used to complement the expression of this system in RGP4. The insertion of this region may have been facilitated by the action of transposases which were found in this region (KP32241, KP32243).

RGP6 has a similar organization to that of the previously described T6SS locus-III identified in strains MGH 78578, NTUH-K2044 and 342 [11] with which the Kp13 region shares, respectively, 95%, 85% and 47% coverage (compared using BLASTN). The presence of this T6SS locus in all compared strains serves as evidence that this region might have been transferred early during the *K. pneumoniae* evolution and have since suffered reorganization within the host chromosomes. Similarly to the T6SS locus-III in strain MGH 78578, this T6SS segment also harbors bacteriocin genes whose predicted protein sequence contain pyocin/cloacin domains (IPR016128) such as KP01496 and KP01501/KP01504, although the latter are found truncated due to the insertion of an IS family 3 belonging transposase. CDSs with a possible immunity role against self bacteriocins are also found in this RGP (KP01494, KP01495, KP01500). Thus, the T6SS found in RGP6 could act conferring Kp13 an advantage on competing with related bacteria through the synthesis and export of bacteriocins.

RGP8 is the third T6SS -related RGP found in the Kp13 chromosome and corresponds to T6SS locus-I described by Sarris *et al*. for other *Klebsiella* spp. [11]. This region is very conserved among the compared strains specially at the initial portion comprising ImpB/COG3516 (KP03060) up to VgrG (KP04343) as well as at the final portion. The central part is variably conserved and in Kp13 there are multiple lipoproteins

(KP04345-KP04347) which have signal-peptide as well as an uncharacterized conserved domain (IPR021733). Other T6SS CDSs detected in this region include IcmF (KP04352), ImpA (KP04353), ImpG (KP04354), ImpH (KP04355) and SciN (KP04356) which were identified based on conserved InterPro and COG domains. A ClpV homolog (KP04342) was also found in this t6ss locus. No mobile genetic elements were identified in the Kp13 RGP, although they occur in clinical isolates MGH 78578 and NTUH-K2044, an indication that genomic reorganization took place within these segments.

RGP10

This plasticity region in Kp13 and related bacteria was identified mainly based on its syntenic break relative to the chromosome of *K. pneumoniae* 342, as well as the presence of a flanking transposase (KP04179). This region is found in all compared strains except for 342, and its gene composition include CDSs coding for hydrolases that could play a role in L-amino acids formation (KP04171, KP04172). An CDS showing 74% amino acid identity to diaminopropionate ammonia lyase from *Salmonella typhimurium* [Swiss-Prot:P40817] is also found in this region (KP04174). An HTH-type regulator (KP04175) located in the opposite strand to the genes supposedly responsible for the enzymatic activities is present, as well as a possible transporter (serine transporter, KP04173). The presence of these genes might indicate a possible orchestrated role for this region that could provide Kp13 and related *K. pneumoniae* (but not strain 342) with metabolic distinctness. Adjacent to the transposase is an CDS (KP04178) annotated as related to β-lactamases due to a conserved domain, although direct evidence is needed in order to confirm whether in fact this gene is expressed and the properties of its product.

RGP11

As some of the previously identified RGPs, this region also has a syntenic break relative to the *K. pneumoniae* 342 chromosome. An insertion sequence is found in this region (KP31763), as well as CDSs with a possible role in sugar transport possibly belonging to the phosphotransferase system family (KP03465-KP03468). Adjacent to these CDSs two sequences with sugar isomerase domains were detected (KP03469, KP03470). It is known that the use of alternative carbon sources represents a selective survival advantage to bacteria, but domain analyses solely do not allow for inferences on the exact carbohydrate that could be transported by these proteins.

RGP12

The insertion of this region occurs near a tRNA for threonine which is immediately followed by an CDS coding for a phage integrase family protein (KP02143) having 91% identity with a sequence from *E. coli* SMS-3-5 (BLASTN, [GenBank:ACB17891]). Multiple transposases are present throughout the region and reveal a complex mosaic structure with Kp13-specific segments as well as shared regions between the compared strains. CDSs coding for fimbrial proteins and adhesins, specific to Kp13, are found in RGP12 (KP02150-KP02154). During the infection process, adhesins bind to specific molecules of the host and thus have been proposed as vaccine targets [12]. The region where RGP12 was inserted may serve as recombination spot in *K. pneumoniae* since in the other compared bacteria the region following the flanking tRNA contain different insertions having only few conserved segments. For instance, in MGH 78578 there is a 25 kbp insertion, while in NTUH-K2044 and 342 the length of these insertions are, respectively, of 23 and 60 kbp (counting from the conserved flanking genes *proA* up to *csiD*). At the 3' terminal of RGP12 we found the

aco operon (*acoKABCD*, KP31250; KP02898-KP02901) involved in acetoin catabolism, this compound being the product of the fermentative metabolism of many microorganisms [13]. These genes are found in Kp13, NTUH-K2044 and 342, but not in MGH 78578, which raises the question of whether the specific segment where *aco* is located was lost in that strain or simply not acquired through HGT events.

RGP13

Similar to the previously described region, the insertion of RGP13 occurs after a tRNA (for leucine), also followed by an CDS with similarity to phage integrases.(KP32364). Most of the CDSs identified in this region are unique to Kp13 relative to the compared strains, and include an acid phosphatase (KP01195) followed by a membrane protein (KP01196) which carries a porin domain (InterPro accession IPR001702). This protein shares 35% identity with the outer-membrane cytotoxic protein ComP from *Pleiomonas shigelloides* (BLASTP, [SwissProt:A0JCJ5]), which was described as able to induce apoptosis in human cells [14]. Multiple ABC-related transporter proteins are found in this RGP, and their coding genes are KP01205, KP32165, KP01200 and KP01201. Also detected were many hypothetical genes, restriction enzymes, insertion sequences and transposases, and it is probable that this region suffered extensive structural reorganization.

References

1. Pieper DH, Martins dos Santos VAP, Golyshin PN (2004) Genomic and mechanistic insights into the biodegradation of organic pollutants. Current Opinion in Biotechnology 15: 215–224. doi:10.1016/j.copbio.2004.03.008.

2. Molina-Henares AJ, Krell T, Eugenia Guazzaroni M, Segura A, Ramos JL (2006) Members of the IclR family of bacterial transcriptional regulators function as activators and/or repressors. FEMS Microbiology Reviews 30: 157–186. doi:10.1111/j.1574-6976.2005.00008.x.

3. Kropinski AM, Kovalyova I V, Billington SJ, Patrick AN, Butts BD, et al. (2007) The genome of epsilon15, a serotype-converting, Group E1 Salmonella enterica-specific bacteriophage. Virology 369: 234–244. doi:10.1016/j.virol.2007.07.027.

4. Fouts DE, Tyler HL, DeBoy RT, Daugherty S, Ren Q, et al. (2008) Complete genome sequence of the N2-fixing broad host range endophyte Klebsiella pneumoniae 342 and virulence predictions verified in mice. PLoS Genetics 4: e1000141. doi:10.1371/journal.pgen.1000141.

5. Cunningham L, Pitt M, Williams HD (1997) The cioAB genes from Pseudomonas aeruginosa code for a novel cyanide-insensitive terminal oxidase related to the cytochrome bd quinol oxidases. Molecular Microbiology 24: 579–591.

6. Hill C, Gray J, Brody H (1989) Use of the isocitrate dehydrogenase structural gene for attachment of e14 in Escherichia coli K-12. Journal of Bacteriology 171: 4083–4084.

7. Wang F, Whittam T, Selander RK (1997) Evolutionary genetics of the isocitrate dehydrogenase gene (icd) in Escherichia coli and Salmonella enterica. Journal of Bacteriology 179: 6551–6559.

8. Bönemann G, Pietrosiuk A, Mogk A (2010) Tubules and donuts: a type VI secretion story. Molecular Microbiology 76: 815–821. doi:10.1111/j.1365-2958.2010.07171.x.

9. Bingle LE, Bailey CM, Pallen MJ (2008) Type VI secretion: a beginner’s guide. Current Opinion in Microbiology 11: 3–8. doi:10.1016/j.mib.2008.01.006.

10. Mougous JD, Gifford CA, Ramsdell TL, Mekalanos JJ (2007) Threonine phosphorylation post-translationally regulates protein secretion in Pseudomonas aeruginosa. Nature Cell Biology 9: 797–803. doi:10.1038/ncb1605.

11. Sarris PF, Zoumadakis C, Panopoulos NJ, Scoulica E V (2011) Distribution of the putative type VI secretion system core genes in Klebsiella spp. Infection, Genetics and Evolution 11: 157–166. doi:10.1016/j.meegid.2010.09.006.

12. Wizemann TM, Adamou JE, Langermann S (1999) Adhesins as Targets for Vaccine Development. Emerging Infectious Diseases 5: 395–403.

13. Deng WL, Chang HY, Peng HL, Pengi H (1994) Acetoin catabolic system of Klebsiella pneumoniae CG43: sequence, expression, and organization of the aco operon. Journal of Bacteriology 176: 3527–3535.

14. Tsugawa H, Ogawa A, Takehara S, Kimura M, Okawa Y (2008) Primary structure and function of a cytotoxic outer-membrane protein (ComP) of Plesiomonas shigelloides. FEMS Microbiology Letters 281: 10–16. doi:10.1111/j.1574-6968.2007.01041.x.
